# Nanoengineering Metal–Organic Frameworks and Derivatives for Electrosynthesis of Ammonia

**DOI:** 10.1007/s40820-023-01169-4

**Published:** 2023-08-24

**Authors:** Daming Feng, Lixue Zhou, Timothy J. White, Anthony K. Cheetham, Tianyi Ma, Fengxia Wei

**Affiliations:** 1 College of Chemistry, Liaoning University, Shenyang, 110036 People’s Republic of China; 2 School of Materials Science and Engineering, Nanyang Technological University, 50 Nanyang Avenue, Singapore, 639798 Singapore; 3 Materials Research Laboratory, University of California, Santa Barbara, CA 93106 USA; 4 School of Science, RMIT University, Melbourne, VIC 3000 Australia; 5 Institute of Materials Research and Engineering (IMRE), Agency for Science, Technology and Research (A*STAR), 2 Fusionopolis Way, Innovis 08-03, Singapore, 138634 Singapore

**Keywords:** Metal–organic frameworks, Electrosynthesis of ammonia, Nitrogen reduction reactions, Nitrate reduction reactions

## Abstract

Electrocatalytic synthesis under mild conditions has become increasingly important as one of the practical alternatives for industrial applications, especially for the green ammonia (NH_3_) industry. A properly engineered electrocatalyst plays a vital role in the realization of superior catalytic performance. Among various types of promising nanomaterials, metal–organic frameworks (MOFs) are competitive candidates for developing efficient electrocatalytic NH_3_ synthesis from simple nitrogen-containing molecules or ions, such as N_2_ and NO_3_^−^. In this review, recent advances in the development of electrocatalysts derived from MOFs for the electrosynthesis of NH_3_ are collected, categorized, and discussed, including their application in the N_2_ reduction reaction (NRR) and the NO_3_^−^ reduction reaction (NO_3_RR). Firstly, the fundamental principles are illustrated, such as plausible mechanisms of NH_3_ generation from N_2_ and NO_3_^−^, the apparatus of corresponding electrocatalysis, parameters for evaluation of reaction efficiency, and detection methods of yielding NH_3_. Then, the electrocatalysts for NRR processes are discussed in detail, including pristine MOFs, MOF-hybrids, MOF-derived N-doped porous carbons, single atomic catalysts from pyrolysis of MOFs, and other MOF-related materials. Subsequently, MOF-related NO_3_RR processes are also listed and discussed. Finally, the existing challenges and prospects for the rational design and fabrication of electrocatalysts from MOFs for electrochemical NH_3_ synthesis are presented, such as the evolution of investigation methods with artificial intelligence, innovation in synthetic methods of MOF-related catalysts, advancement of characterization techniques, and extended electrocatalytic reactions.

## Highlights

- Recent advances in the metal–organic framework (MOF)-related catalysts for electrochemical ammonia synthesis protocols under ambient reaction conditions are summarized and discussed.
- The design and fabrication of efficient electrocatalysts from MOF for the reduction of N_2_ and NO_3_^−^ are systematically analyzed.
- Based on the current advances, the ongoing challenges and promising perspectives are highlighted.

## Introduction

Ammonia, which has been applied in fertilizers to protect the world’s population from the global food crisis in the twentieth century, may save the world again from today’s climate change crisis by serving as a green fuel. However, the current NH_3_ industry, which uses a catalytic conversion from N_2_ and H_2_ at high temperatures and pressures (the Haber–Bosch process), is far from green [1]. Consequently, the production of green ammonia under mild conditions has become a major focus in low-carbon, green energy research.

Heterogeneous catalysis has attracted extensive attention in the fields of chemistry [2], energy [3], environmental protection [4], and so forth [5]. Among all the catalytic processes, the electrocatalytic transformation of abundant small molecules or ions (N_2_ and NO_*x*_) into value-added products, such as NH_3_, has been considered a very promising alternative to the current industrial methodologies and a smart approach to tackling intractable environmental issues and the energy crisis [6]. With renewable energy as the power supply, transportable fuels (e.g., NH_3_) and chemicals that are obtained from renewable feedstocks (e.g., H_2_ from water splitting, N_2_) can be tuned by varying the bias potential under ambient conditions. In addition, large-scale industrial applications might be facilitated by using compact and modular electrochemical reactors. However, these electrochemical conversions are limited by the unavoidably large overpotentials for the adsorption and bond cleavage of the intrinsically inert small molecules and the migration of multiple electrons that are required by the subsequential reduction and possible coupling reactions. Furthermore, the competing hydrogen evolution reaction (HER) simultaneously occurs in aqueous electrolytic solutions, impairing the catalytic selectivity and faradaic efficiency (FE) of the electrocatalytic conversion of simple molecules [7]. To overcome the kinetic barriers and sluggish reaction dynamics, diverse approaches have been employed to precisely construct nanomaterials with specific electrocatalysis functions.

In general, the diffusion of substrates and products, the transfer of electrons, and reactions at the electrode/electrolytic solution interface are three critical sections of a typical electrocatalytic process [8]. Therefore, the transport of charges and the catalytic capability of active sites strongly influence the resulting performance of the catalysts. Consequently, maximum catalytic reactivity can be achieved by modulating the electronic structure and manipulating the surface of the catalytic materials, mimicking homogeneous catalysis in microscopic reaction environments.

Due to their special metal–ligand, periodic networks with metal ions/cluster as nodes and organic ligands as linkers, metal–organic frameworks (MOFs) with dispersed monoatomic active sites exhibit unique quasi-molecular catalytic performance in heterogeneous catalysis [9]. Permanent porosity and chemical and thermal stability of the crystalline MOF networks can be ensured by strong metal–ligand bonds. By judicious selection of the building blocks and appropriate reaction conditions, it is possible to achieve rational design and engineering of the frameworks. Owing to their controllable morphology, high surface area, designable porosity, tunable pore size, and flexible electronic structure, MOFs have not only exhibited extraordinary performance in gas storage and separation [10, 11], energy storage [12–16], microwave adsorption [17–19], sensors [20, 21] and magnetic and molecular recognition [22] but have also received extensive attention to directly activate and convert kinetically inert, simple molecules [23, 24] over the last few years. In addition to their direct use as electrocatalysts, hybrid supports, or functional tuners, MOF materials can also be used as electrocatalyst precursors or templates for further fabrication of conductive and stable nanoporous carbon materials (NPC) [25]. The subsequent pyrolysis of the MOFs under various conditions can lead to the formation of highly dispersed nanoparticles or single-atom catalysts (SACs), resulting in enhanced electrocatalytic performance [26].

In recent years, the progress of MOF-based catalysts has been demonstrated by many experimental results, and several comprehensive reviews have been published in the field of electrocatalysis. For example, Zhang and co-workers summarized the progress of MOF-derived electrocatalytic materials, focusing on the characterization of active catalytic sites, catalytic performance, and mechanisms in various types of electrochemical catalysis, such as HER, oxygen evolution reaction (OER), oxygen reduction reaction (ORR), CO_2_RR, and NRR [27]. Meanwhile, Xu and colleagues reported on recent advances in the fabrication of MOF-derived SACs and their applications in electrocatalysis [28]. Li and co-workers demonstrated the similarities shared by CO_2_RR and NRR catalyzed by earth-abundant electrocatalysts (including MOFs), such as the intrinsic chemical inertness of CO_2_ and N_2_, the multiple-electron transfer process with slow kinetics for reduction, and the competing HER as a side reaction [29]. Meanwhile, Tang and Ge reviewed the recent advances of MOF-related materials in the CO_2_RR process [30]. Huo and Zhang suggested that the defects in MOFs are the main features to enhance the NRR performance [31]. Since ammonia synthesis is of great importance in the development of modern civilization, we envisaged that a comprehensive review of the electrosynthesis of NH_3_ by MOF-related catalysts would be meaningful and timely [32].

With our continuous interest in the electrochemical synthesis of NH_3_ [33–37], this review presents a systematic discussion of the MOFs and MOF-derived materials that are being studied for the electrosynthesis of ammonia from N_2_ and NO_3_^−^. First, the fundamental principles of the electrosynthesis of NH_3_ are illustrated. Then, the recent works related to this topic are divided into four main parts according to the role played by the MOFs in the electrocatalytic process. Finally, the existing challenges and main drawbacks of MOF-related electrocatalysts, as well as promising future solutions and new paths, are analyzed and proposed. The examples of recent work are mainly collected from 2017 onwards.

## Fundamental Principles for Electrosynthesis of NH_3_

### Reaction Mechanisms

#### Reduction of Dinitrogen Gas

Following recent advances in mild electrochemical NRR, plausible catalytic mechanisms have been proposed based on theoretical and experimental analysis, which not only provide clear clues for understanding the actual reaction pathway but also offer guidance for the rational fabrication of efficient and robust electrocatalysts. The electrocatalytic NRR process generally consists of three main steps: (1) the adsorption and activation of N_2_ on the catalytic sites; (2) the hydrogenation process of the activated N_2_ intermediates; (3) the desorption of NH_3_ from the catalytic sites.

**Fig. 1:**
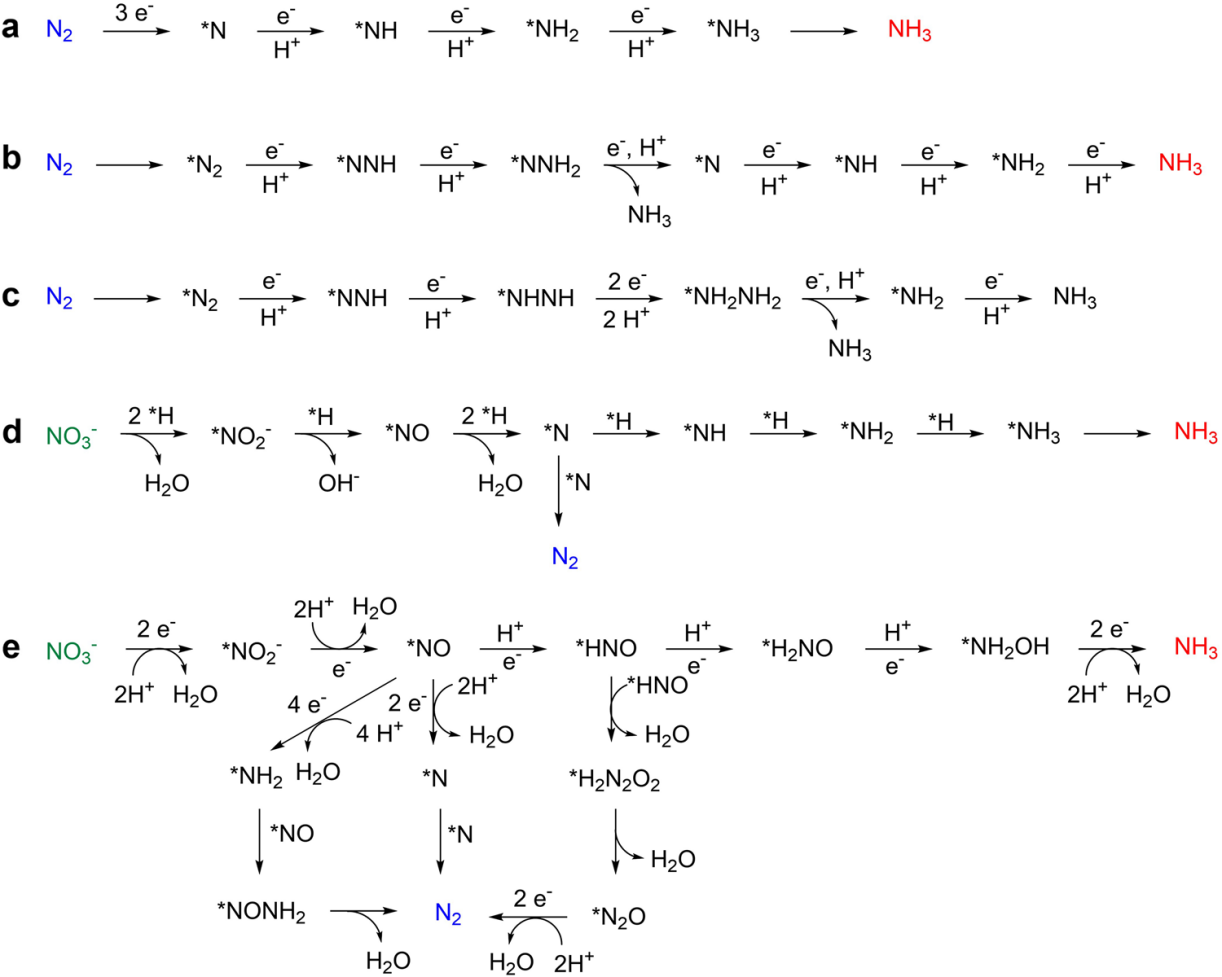
Plausible mechanisms for electrosynthesis of NH_3_. **a** Dissociative pathway for NRR. **b** Associative distal pathway for NRR. **c** Associative alternating pathway for NRR. **d** Adsorbed-hydrogen-mediated pathway for NO_3_RR. **e** Direct electroreduction of NO_3_RR

For MOF-based heterogeneous electrocatalysis, two major NRR mechanisms are proposed in accordance with the protonation and bond-breaking modes of *N_2_ intermediate, including associative and dissociative pathways. In the dissociative pathway, the cleavage of the N≡N bond takes place upon the adsorption of the N_2_ molecule (Fig. 1a) [38]. After three consecutive protonation, the NH_3_ molecule is generated and further released from the catalytic site. It is worth mentioning that the bond-breaking process requires extremely high energy, due to which the Haber–Bosch process has to be conducted under harsh conditions, thus not popular in electrocatalysis under mild conditions. In the associative pathway, the bond cleavage proceeds with the protonation process simultaneously, yielding a series of N_*x*_H_y_ intermediates. In accordance with the order of hydrogenation, the associative path is further divided into distal and alternating routes. In the distal path, the protonation and reduction happen on the distal nitrogen atom first (Fig. 1b) [39]. After the generation and release of distant NH_3_, the nitrogen atom bound on the catalyst surface begins to form the second NH_3_ in a similar route. Regarding the alternative path, protons are introduced successively onto the two nitrogen atoms and the two NH_3_ molecules are formed in turn (Fig. 1c) [40].

#### Reduction of Nitrate Ion

In the electrochemical reduction of nitrate ions, there are two observed products so far, i.e., N_2_ and NH_3_, and the possible mechanistic pathway are complicated, including multiple-electron-transfer and diverse nitrogen-containing intermediates from + 5 to − 3 valence states [41]. Theoretical and experimental results suggest two synthetic routes for NH_3_ production: regulation by binding hydrogen atoms and direct reduction from the cathode. About adsorbed-hydrogen-mediated pathway, the nitrate ion is subsequently and continuously reduced by the in situ generated *H from pre-reduction of *H_2_O on the catalyst surface (Fig. 1d) [42]. The desired NH_3_ is produced through a series of intermediates, such as *NO_2_^−^, *NO, *N, *NH, *NH_2,_ and so on. It is worth mentioning that the N_2_ may be formed through the combination of two *N intermediate. Since the calculated migration barrier of *H is less than that of *N and N–H bond formation is kinetically more favorable than the N–N bond, the preferential generation of NH_3_ can be achieved on more photophilic catalytic sites. Regarding the electron-mediated pathway, the NO_3_^−^ is adsorbed and reduced to *NO_2_ and *NO successively (Fig. 1e) [43]. Subsequently, the NH_3_ is obtained through consecutive hydrogenation of *NO intermediate, experiencing *HNO, *H_2_NO, *NH_2_OH, and so on. In addition to the formation of NH_3_, the possible N_2_ product may be provided through N–N coupling between intermediates, such as *NO and *NO, *NO and *NH_2_, *N and *N, *HNO and *HNO, and so forth.

### Evaluation of Efficiency

#### Nitrogen Efficiency (NE)

The NE is calculated to evaluate the utilization efficiency of the nitrogen atom in the conversion of nitrogen-containing molecules, especially in the transformation of soluble nitrogen oxides, such as nitrate and nitrite ions. The magnitude is determined by the ratio between the actual amount of nitrogen atoms in the product and those in the reactants. It is notable that due to the insolubility of N_2_ gas and NO gas, the concentrations of N_2_ and NO are uncertain during the experiments, this parameter cannot be applied in the reduction of N_2_ and NO processes. An example equation is shown in the following for the evaluation of NO_*x*_^−^ reductions:

1 $$ \begin{array}{*{20}c} {{\text{NE}} = \frac{{\left[ {{\text{NH}}_{3} } \right]}}{{\left[ {{\text{NO}}_{x}^{ - } } \right]}} \times 100\% } \\ \end{array} $$

where [NO_*x*_^*−*^] is the concentration of soluble nitrogen oxides (nitrite ion when *x* = 2, nitrate ion when *x* = 3) in a pre-reaction solution (mol L^−1^), [NH_*3*_] is the resulting NH_3_ concentration (mol L^−1^).

#### Faradaic Efficiency (FE)

The FE is the parameter for the evaluation of the efficiency of electric charges in electrocatalysis. The higher the FE value presented; the more electrons are effective in the generation of desired products. The following equation is generally adopted for FE calculation in the electrosynthesis of NH_3_:

2 $$ \begin{array}{*{20}c} {{\text{FE}} = \frac{{n \times F \times \left[ {{\text{NH}}_{3} } \right] \times V}}{Q} \times 100\% } \\ \end{array} $$

where *n* is the number of electrons required to generate one NH_3_ molecule from substrates (*n*_NRR_ = 3, *n*_NO3RR_ = 8), *F* is Faraday constant (96,485 C mol^−1^), [NH_*3*_] is the resulting NH_3_ concentration (mol L^−1^), *V* is the volume of the electrolyte in the cathode chamber (L), and *Q* is the total charges passed through the electrode (C).

#### Ammonia Yield Rate

The ammonia yield rate, also simplified as NH_3_ yield, is used to evaluate the catalytic activity and stability. The larger magnitude means a greater amount of NH_3_ produced per unit of time per unit area. The following equation is commonly used:

3 $$ \begin{array}{*{20}c} {{\text{NH}}_{3} {\text{Yield}}\, {\text{Rate}} = \frac{{\left[ {{\text{NH}}_{3} } \right] \times V}}{t \times S}} \\ \end{array} $$

where [NH_3_] is the resulting NH_3_ concentration (mol L^−1^), *V* is the electrolyte solution volume in the cathodic chamber (L), *t* is the NRR time in total, and *S* is the area of the working electrode (i.e., 2 × 2 cm^2^).

### Detection of Ammonia

The current technologies for electrosynthesis of NH_3_ in mild conditions are not yet ready for practical use. One of the biggest obstacles is only a trace amount of NH_3_, or NH_4_^+^ is present in the resulting aqueous electrolytic solution. Therefore, the accurate detection of NH_3_ at low concentrations is crucial for the development of technological routes for electrochemical NH_3_ generation. To date, a number of detection protocols have been proposed, which can be divided into two main categories—the colorimetric method and the instrumental analysis method [44].

#### Colorimetric Methods

By measuring and comparing the resulting electrolytic solution, the colorimetric methods are rapid, accurate, and reliable for detecting the concentration of NH_3_, especially at trace levels. Generally, certain reagents can change the color of the NH_3_-containing solution. Pretreatment of the electrolytic solution is required to minimize interference from other ions, so ultrapure water and pre-cleaned apparatus are essential when carrying out experiments. Besides, a standard calibration curve is also needed. The following contents are an introduction to the two common colorimetric methods in more detail: (1) The Nessler’s reagent, a solution containing K_2_HgI_4_ and KOH, is used in Nessler’s method for quantitative detection of NH_3_. In alkaline solutions, the mercury and iodide ions react with NH_3_, which is almost unaffected by the pH value of the testing sample. The resulting reddish brown complex shows a strong absorbance at 420 nm, which is directly proportional to the concentration of NH_3_ in a relatively wide range. However, due to the toxic nature of the mercury ions, careful manipulation of the detection is required. (2) The indophenol blue method (IB) is the reaction of NH_3_ with phenol and hypochlorite under alkaline conditions, which is known as the Berthelot reaction. Catalyzed by sodium nitroprusside, the blue indophenol product can be obtained at the end of the reaction, and the intensified color change can be detected by UV–Vis absorption. Notably, a relatively long reaction time is essential, and overestimation happens with a higher concentration of NH_3_.

#### Instrumental Analysis Methods

Generally, the instrumental analytical methods are more efficient, convenient, sensitive, and stable than the colorimetric methods. And the resulting electrolytic solution can be directly tested without the pre-treatment. The following are examples that have ever been reported for the detection of NH_3_. (1) Ion chromatography (IC) is one of the high-performance liquid chromatography, which can quantitatively detect NH_4_^+^ with acidic eluents and cationic columns in aqueous solutions at a trace level. As the ammonium ion has a similar retention time to the sodium and potassium ions, the results are susceptible to their influences. Therefore, this technique suffers from limited choices of electrolytes. (2) An ammonia-sensitive electrode (ASE) is a combination of a pH electrode and an ammonia sensor. After alkalizing the sample solution, the NH_3_ can be detected by ASE when it escapes from the solution and subsequently affects the magnitude of the electric current. Subsequently, the concentration of NH_3_ can be obtained after calculation and comparison with a pre-calibrated standard curve. (3) Proton nuclear magnetic resonance (^1^HNMR) is generally applied for the characterization of organic molecules, which also can determine the NH_3_ qualitatively and quantitatively. A high-resolution NMR instrument and water-eliminated Fourier transform technique can effectively monitor the NH_4_^+^ in an aqueous solution. The amount of the ions can be calculated by the internal standard method. Furthermore, the nitrogen source can also be confirmed through isotopic experiments.

### Electrolysis Apparatus

**Fig. 2:**
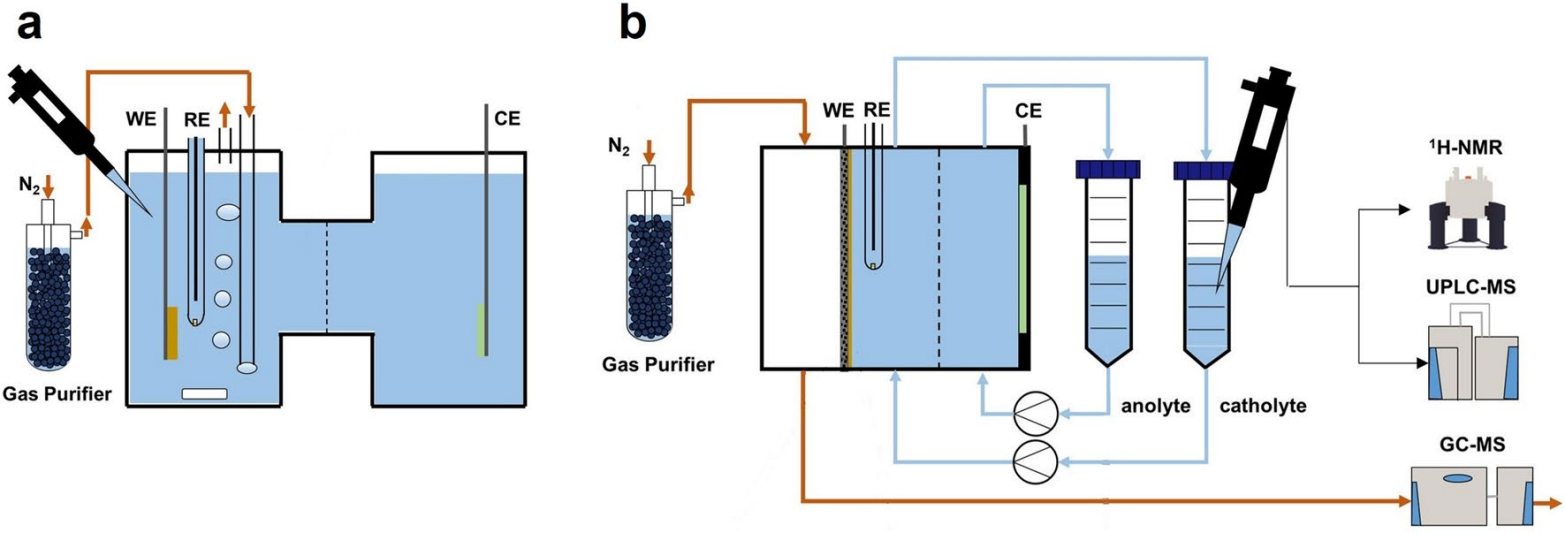
**a** An H-type cell in NRR. **b** A flow cell in NRR with detection methods. Reproduced with permission from Ref. [45]. Copyright 2022, American Chemical Society

As one of the vital impact factors for efficient electrocatalysis, various electrolysis cells have also been investigated by researchers. To date, two typical types of electrolysis cells were widely used in the electrochemical synthesis of ammonia, including H-type cells and flow cells [45]. A typical H-type cell is composed of a cathode chamber for oxidation and an anode chamber for reduction, which is separated by a proton/ion-exchange membrane to prevent the bypass of products but maintain the overall conductivity (Fig. 2a). In an H-type cell, the reduction reaction can be severely influenced by competing HER from water splitting and limited mass transportation in bulk aqueous solution, while in bulk organic solution, the decrease in electric conductivity is an indispensable challenge for sufficient electrolytic performance. Meanwhile, the aforementioned drawbacks of H-type cells can be mitigated by a flow cell. Composed of separated gaseous and catholyte cathode chamber, generally in the form of a gas diffusion electrode (GDE), the reduction reactions take place in the triple-phase boundary, within which much higher current densities and enhanced efficiencies can be obtained (Fig. 2b).

## MOF-related Electrocatalysts for Nitrogen Reduction Reaction

As a naturally abundant nitrogen resource, dinitrogen gas is one of the ideal resources for the synthesis of NH_3_. However, due to the inert nature of N_2_, the transformation from N_2_ to NH_3_ remains challenging. Electrochemical fixation of N_2_ into NH_3_ is one of the promising alternatives to replace the energy-intensive Haber–Bosch process. With the aid of electrocatalysts, the N_2_ molecules can be adsorbed, activated, and transformed into NH_3_. Plenty of efforts have been taken into developing novel catalysts with efficient and robust performance for the electrosynthesis of NH_3_ under ambient conditions. The following context mainly focused on the artificial electrochemical conversion of N_2_ to NH_3_ catalyzed by MOFs and MOF-derived materials.

The design principles for highly efficient MOF-related electrocatalysts for NRR follow but are not limited to:

1. Modifying the electronic structures to enhance N_2_ adsorption and activation, or altering the NRR pathways, such as doping, hybridization, or bimetallic MOFs, etc.
2. Tailoring the porous framework to improve mass transport, e.g., ligand engineering, etc.
3. Enhancing the number of active sites, e.g., increasing the under-coordinated metal sites, or creating ion vacancies, etc.
4. Increasing conductivity to enhance charge transport, such as doping or metal species alteration, etc.
5. Tuning the surface hydrophobicity to suppress the competing HER, e.g., via organic ligands optimization, surface treatment, etc.
6. Hybridizing with other species for synergistic electrocatalytic effects.
7. Manipulating the exposed surface condition of the catalyst, such as particle size to enlarge the effective surface area and the crystal orientations to uncover the highly active crystal surfaces, e.g., through the optimization of synthetic strategies, etc.
8. Optimizing the electrolytes to favor the NRR process, including choices of electrolytes, pH, etc.

### MOF-Based Electrocatalysts

With metal nodes and organic linkage, MOFs are recognized as the ideal catalysts that can be precisely manipulated for specific catalytic capacity. Moreover, due to the large surface area and amendable organic functional groups, enhanced catalytic performance can be achieved [46]. Furthermore, hybrid with additional catalytic materials, such as pure metallic plates, metal oxides [47], metal chalcogenides [48], MXene [49], graphite [50], and so on, MOFs can tune the reaction pathway and subsequently produce targeted products with their intrinsic hydrophobicity and porous structure. The following contents are mainly focused on the pristine MOFs and MOF-hybrid composites for electrocatalytic N_2_ fixation.

#### Monometallic MOFs

To date, various monometallic MOFs, including Al, Fe, Co, Cu, Zr, Ce, In, and so on, exhibited their effective catalytic performance for the generation of NH_3_ via electrochemical N_2_ fixation. Inspired by the high catalytic activity of Fe in Haber–Bosch process, in 2017 Zhao et al. adopted pristine Fe-MOFs as NRR catalysts [51], which was constructed by the Fe^3+^ and 1,3,5-benzenetricarboxylic acid (BTC) and was found to give the highest NH_3_ yield rate (7.63 × 10^–3^ mmol h^−1^ cm^−2^) with 1.43% FE at 90 °C in N_2_. Due to MOFs’ porous structure and abundant transition metal sites, they can absorb and activate the inert N_2_ for electrosynthesis of NH_3_ under relatively mild conditions. Later on, MIL-88B-Fe and NH_2_-MIL-88B-Fe were further fabricated and applied to the NRR process by the same research group [52]. The -NH_2_ group promotes charge transfer to the Fe unsaturated sites and creates a larger effective area. In a neutral electrolytic solution, NH_2_- substituted Fe-MOF promoted the ammonia yield rate up to 4 times higher than that of the original one, achieving 12.45% FE at 0.05 V versus RHE. Apart from Fe-MOFs, the typical Cu-MOF (HKUST-1) was also investigated as an electrocatalyst for NRR [53]. The unsaturated Cu could not only be used as an adsorbent site but also be converted into Cu_2_O or Cu (0) during the reduction process, which increased the conductivity of MOF and achieved the ammonia yield up to 46.63 µg h^−1^ mg_cat._^−1^ with a 2.45% FE at − 0.75 V versus RHE in 0.1 M Na_2_SO_4_. In another Cu-MOF-catalyzed electrochemical NRR work, Zhao et al. adopted the stable self-support JUC-1000/CC as both cathode and anode in an H electrolytic cell [54]. Instead of the OER process, the oxidation of sodium gluconate took place at the anode, generating glucaric acid as another valuable product. With this kinetically favorable process, the electrochemical NRR at the cathode was enhanced significantly, generating NH_3_ at a high ammonia yield of 24.7 mg h^−1^ mg_cat._^−1^ with 11.9% FE. The introduction of a new reaction into an anode chamber not only increases the diversity of products but also provides a new direction for the improvement of the electrochemical NRR process. Improving the electrical conductivity of MOF is always challenging. In this regard, efforts have been taken to design and fabricate conductive MOFs. For instance, using hexahydroxytriphenylene (HHTP) as organic linkages and cobalt as metal nodes, a conductive Co-MOF (Co_3_HHTP_2_) was constructed for electrocatalytic N_2_ fixation to NH_3_ [55]. In 0.5 M LiClO_4_ electrolytic solution, Co_3_HHTP_2_ nanoparticles achieve a large NH_3_ yield of 22.14 μg h^−1^ mg_cat._^−1^ and a FE of 3.34% at − 0.40 V versus RHE.

**Fig. 3:**
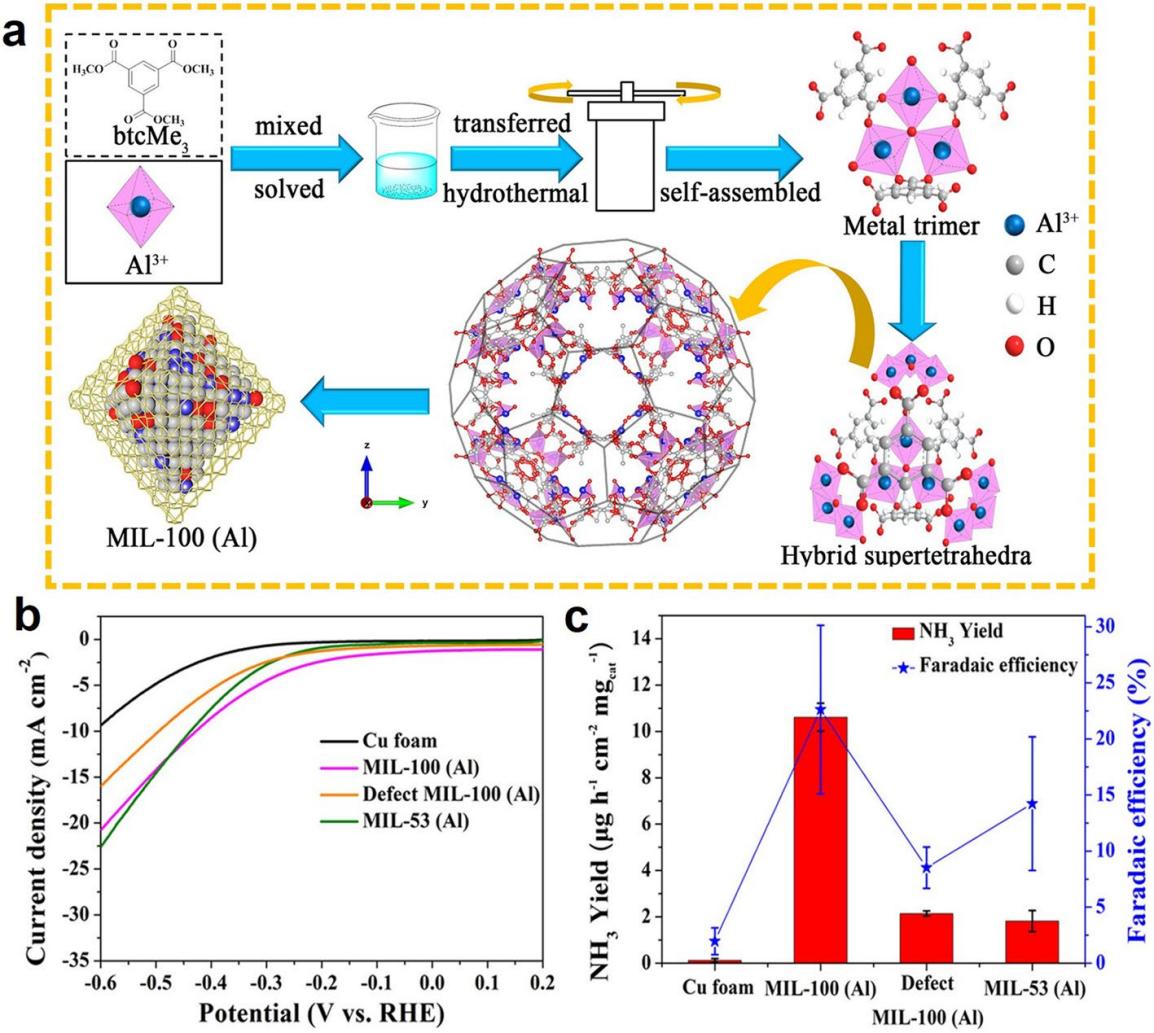
**a** Synthetic strategy for the preparation of MIL-100. **b** Linear sweep voltammetry (LSV) curves and **c** ammonia yield and FE at the overpotential (177 mV) for pure Cu foam, MIL-100(Al), defect MIL-100(Al), and MIL-53(Al) electrodes in an N_2_-saturated aqueous solution of 0.1 M KOH. Reproduced with permission from Ref. [56]. Copyright 2020, American Chemical Society

In recent studies, the main group elements exhibited considerable performance as NRR catalysts due to their effectiveness in suppressing hydrogen evolution, which may originate from the intrinsic affinity of the metal v band to the N 2*p* orbital of N_2_ [33]. For example, Fu et al. first developed an Al-based porous MOF, MIL-100 (Al), for artificial N_2_ fixation through electrocatalysis (Fig. 3a) [56]. Because of the strong interaction between the 3*p* band of Al and the 2*p* orbital of N, MIL-100 (Al) owns excellent ability for N_2_ sorption, presenting remarkable performance in generating NH_3_ with 10.6 µg h^−1^ cm^−2^ mg_cat._^−1^ yield rate and FE of 22.6% at an ultralow overpotential in N_2_-saturated alkaline solution (Fig. 3c). Notably, the synergistic effect of the skeleton and Al nodes of MIL-100(Al) was further revealed by comparative experiments (Fig. 3b). The moderated catalytic performance was observed in the NRR process catalyzed by MIL-53(Al) and defective MIL-100(Al). Furthermore, modified with stable superoxide radicals (O_2_·), a defective Zr-MOF (UiO-66-NH_2_) was applied for electrosynthesis of NH_3_ from N_2_ as a superior active catalyst [57]. In 0.1 M Na_2_SO_4_, the NH_3_ was generated at a high yield rate of 52.81 µg h^−1^ cm^−2^ mg_cat._^−1^ with 85.21% FE at − 0.39 V versus RHE. The neighboring exposed Zr atoms (Zr-OO· and Zr site) contribute to the incredible catalytic performance through their synergy effect. Besides, the proton adsorption was unfavored by the exposed Zr atoms, which further constrained the hydrogen evolution and hydrazine formation.

**Fig. 4:**
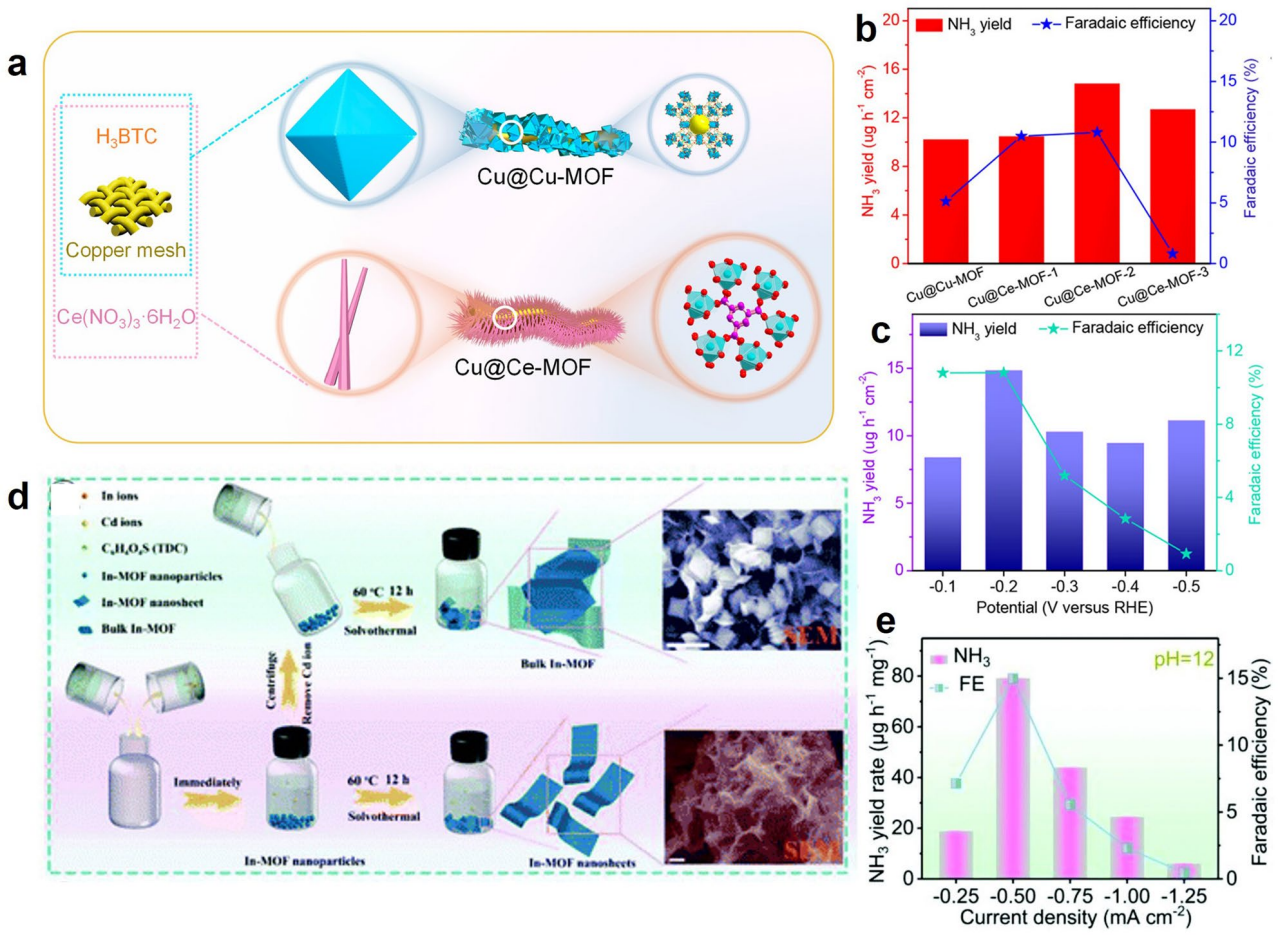
**a** Synthetic diagram of Cu@Cu-MOF and Cu@Ce-MOF. NRR results of **b** different catalysts and **c** Cu@Ce-MOF-2 at different potentials. Reproduced with permission from Ref. [58]. Copyright 2021, American Chemical Society. **d** Synthesis and morphological characterization of In-MOF nanosheets. **e** NRR performance of In-MOFs in pH = 12 aqueous media. Reproduced with permission from Ref. [59]. Copyright 2021, Royal Society of Chemistry

In addition to functionalized MOFs, the in situ generated MOFs on commercially available substrates also presented a noteworthy performance in electrochemical N_2_-to-NH_3_ conversion. For example, Liu et al. constructed several self-support heterogeneous catalysts through in situ growth of MOFs on the copper mesh, including Cu@Cu-MOF and Cu@Ce-MOF-*n* (*n* = 1, 2, and 3) (Fig. 4a) [58]. After a thorough examination, the Cu@Ce-MOF-2 rise above others, and an NH_3_ yield rate of 14.83 µg h^−1^ cm^−2^ with a 10.81% FE was obtained at − 0.2 V versus RHE in 0.1 M KOH (Fig. 4b, c). The Ce-unsaturated coordination structure on the surface of Ce-MOF provides oxygen vacancies and serves as active sites for the adsorption and activation of N_2_, and the conductive copper mesh promotes sufficient charge transfer, making Cu@Ce-MOF-2 an efficient and stable electrocatalyst. However, the poorly stable Ce-MOF reconstructed into CeO_2_ without oxygen vacancies after use.

As an emerging MOF type, two-dimensional (2D) MOF nanosheets show superior catalytic performance due to increased exposed catalytic site, enlarged aspect ratio, enhanced permeability, and improved mass transport. Particularly, Sun and co-workers constructed an ultrathin 2D In-MOF with a thickness of 1.3 nm for the exploration of catalytic performance in NRR at different pH values (1, 7, and 12) (Fig. 4d) [59]. In an alkaline electrolytic solution (pH = 12), the maximum NH_3_ yield of 79.20 µg h^−1^ mg_cat._^−1^ was achieved with a 14.98% FE, while moderate results were obtained in acidic and neutral media (Fig. 4e). In-MOFs possess a rigid architecture and exhibit excellent stability at all pH levels.

**Fig. 5:**
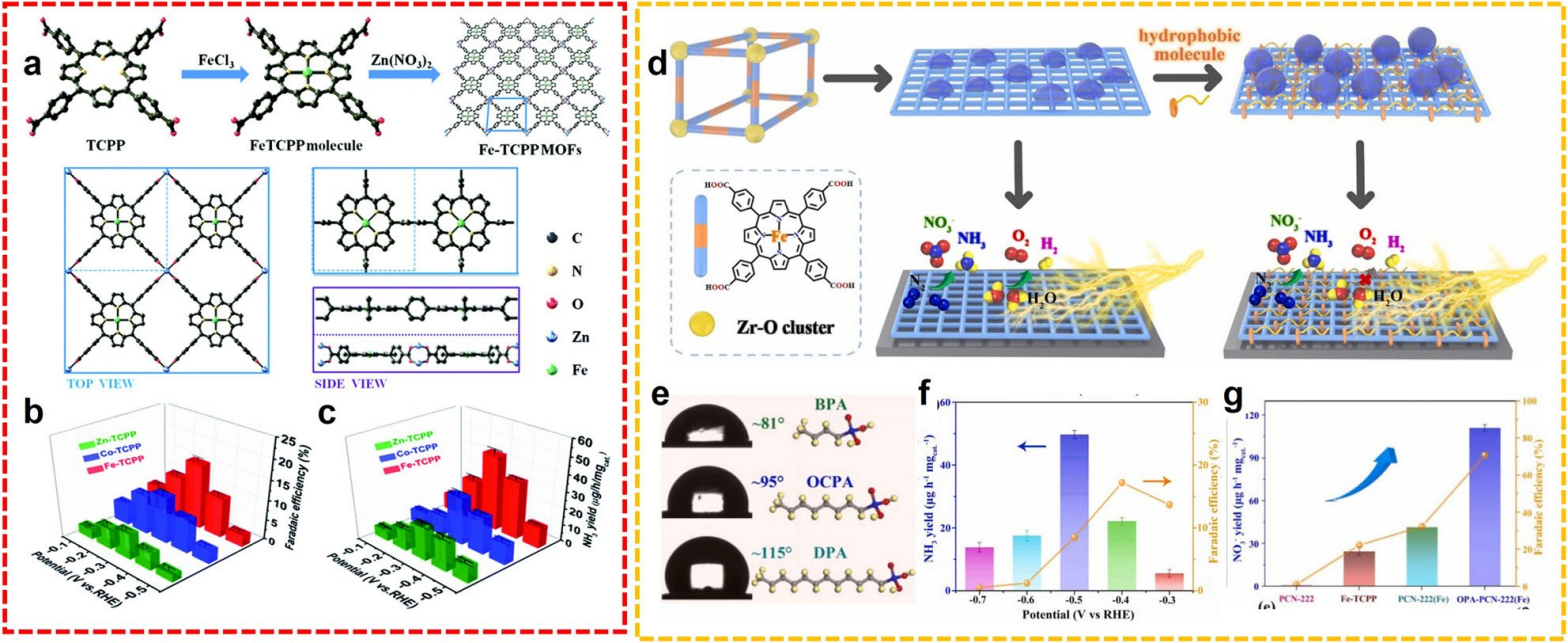
**a** Synthesis of Fe-TCPP MOFs. NRR results of **b** FE and **c** NH_3_ yield of M-TCPP, at different potentials. Reproduced with permission from Ref. [60]. Copyright 2021, Royal Society of Chemistry. **d** Synthetic diagram of hydrophobic PCN-222(Fe) nanosheets for electrocatalytic NRR. **e** Different hydrophobic molecules and water contact angle of modified PCN-222(Fe). NRR performance of **f** OPA-PCN-222(Fe) and **g** different electrocatalysts. Reproduced with permission from Ref. [61]. Copyright 2022, Elsevier

As superior molecular catalysts, metalloporphyrin, and derivatives were commonly adopted as catalytic centers for diverse reactions. Cong et al. installed metalloporphyrin (M-TCPP) motifs into MOFs by assembling with Zn(NO_3_)_2_, constructing thin M-TCPP nanosheets for NH_3_ production (Fig. 5a) [60]. The MOFs with different metal sites were optimized, and Fe-TCPP exhibited a particularly outstanding performance for generating NH_3_. The maximum NH_3_ yield rate (44.77 µg h^−1^ mg_cat._^−1^) and FE (16.23%) were provided at − 0.3 V versus RHE in 0.1 M HCl (Fig. 5b, c). This work integrated the merit of molecular catalysts and 2D nanosheets for promoting the electrochemical conversion of the N_2_ to NH_3_ process. Moreover, another ferriporphyrin-based MOF, PCN-222(Fe) was also synthesized and decorated with hydrophobic molecules, organic phosphoric acids (OPA), for electrocatalytic NRR in 0.1 M HCl (Fig. 5d) [61]. Surface hydrophobicity modification is a viable approach to restrain HER for accelerating N_2_-to-NH_3_ fixation (Fig. 5e). Therefore, the as-fabricated OPA-PCN-222(Fe) demonstrated the generation of NH_3_ with 49.7 µg h^−1^ mg_cat._^−1^ yield rate and 17.2% FE at − 0.5 and − 0.4 V versus RHE, respectively (Fig. 5f). Such catalytic performance was significantly superior to that of PCN-222, Fe-TCPP, and PCN-222(Fe) (Fig. 5g).

#### Bimetallic MOFs

**Fig. 6:**
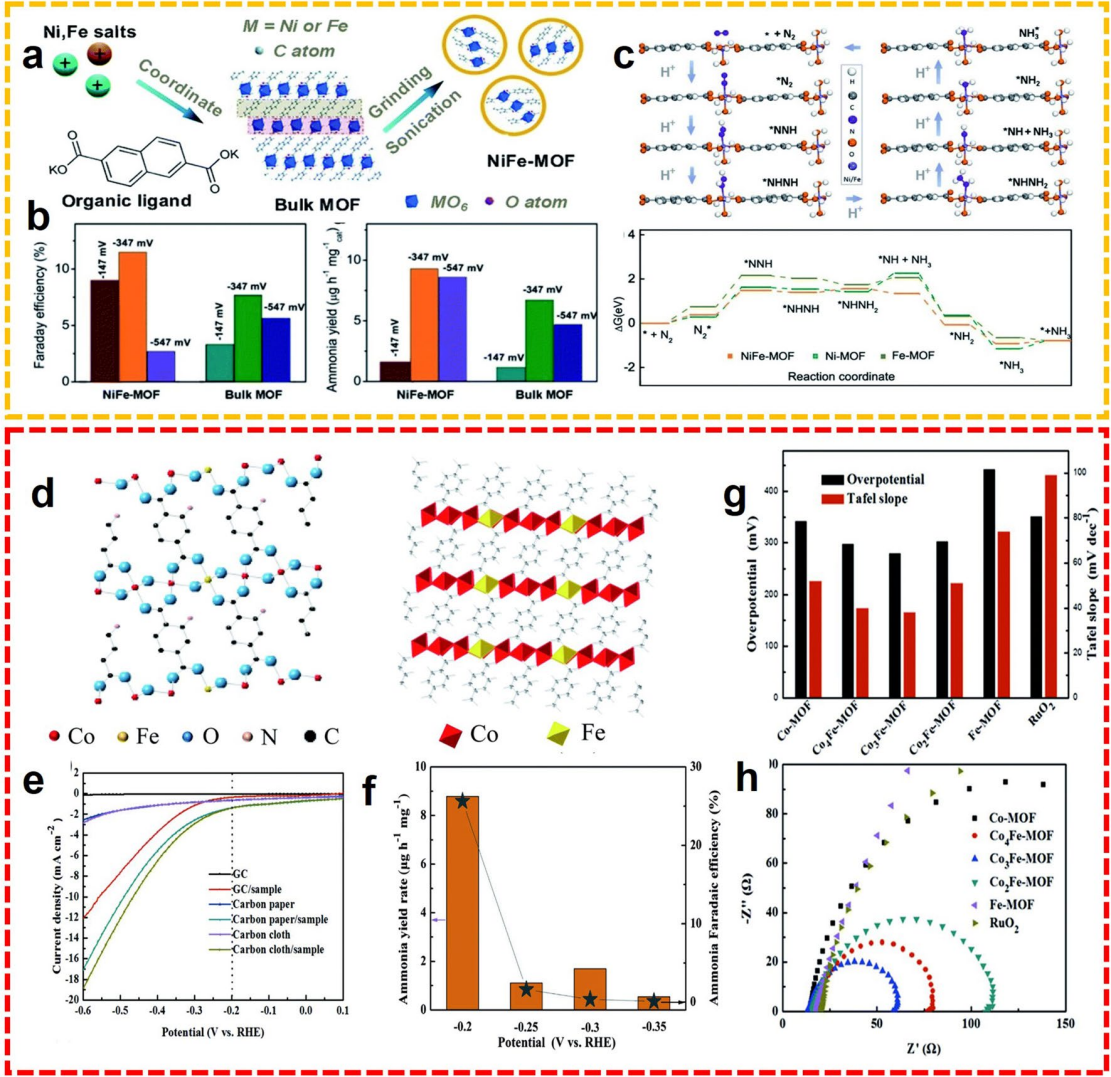
**a** Synthetic strategy of bimetallic NiFe-MOFs. **b** FE and NH_3_ yields of NiFe-MOF and bulk MOF at different potentials. **c** DFT calculations of NiFe-MOF and its counterparts. Reproduced with permission from Ref. [62]. Copyright 2020, Royal Society of Chemistry. **d** Crystal structures of Co_3_Fe-MOF. **e** LSV curves of different working electrodes. **f** NRR properties of Co_3_Fe-MOF. OER electrochemical performances of different working electrodes under ambient conditions **g** comparison on overpotential at 10 mA cm^−2^, Tafel slope, and **h** Nyquist plots. Reproduced with permission from Ref. [63]. Copyright 2020, Royal Society of Chemistry

Recently, it has been shown that the NRR activity of MOF coordinated with only one metal site is relatively insufficient, and the performance of NRR is expected to improve with the introduction of additional metals to tailor the electronic structures. More active sites can also be created. The strong synergistic effect between bimetallic MOF makes it one of the most active catalysts in the field of electrocatalytic NRR. For example, Duan et al. prepared a zero-dimensional (0D) bimetallic nickel, iron-MOF with expanded porosity and the diminished particle size facilitating the mass and charge transport, which further improved the NRR activity (Fig. 6a) [62]. The DFT calculation emphasized that the iron-dopant minimized the Gibbs free energy in the final cleavage of the N–N bond, affording a FE of 11.5% and an ammonia yield rate of 9.3 mg h^−1^ mg_cat_.^−1^ at − 345 mV versus RHE (Fig. 6b, c). Reducing the particle size to expose more active metal sites, increasing mesoporosity to accelerate the mass transport, and modifying the electronic structure to lower the energy barriers for the NRR process, are very efficient to boost the electrocatalytic performance. Additionally, in order to improve the conductivity of MOF, Li et al. developed a 2D bimetallic Co_*x*_Fe MOF nanosheet for the electrosynthesis of NH_3_ (Fig. 6d) [63]. After extensive research, the as-fabricated MOF nanosheet meets the requirements of both OER and NRR, and Co_3_Fe MOF exhibited the best catalytic performance beyond others (Fig. 6e-h). With the aid of this bifunctional electrode, the NH_3_ was generated with a remarkable FE up to 25.64%, and the corresponding NH_3_ yield rate of 8.79 µg h^−1^ mg_cat._^−1^ at − 0.2 V versus RHE.

#### MOF Hybrid Composites

**Fig. 7:**
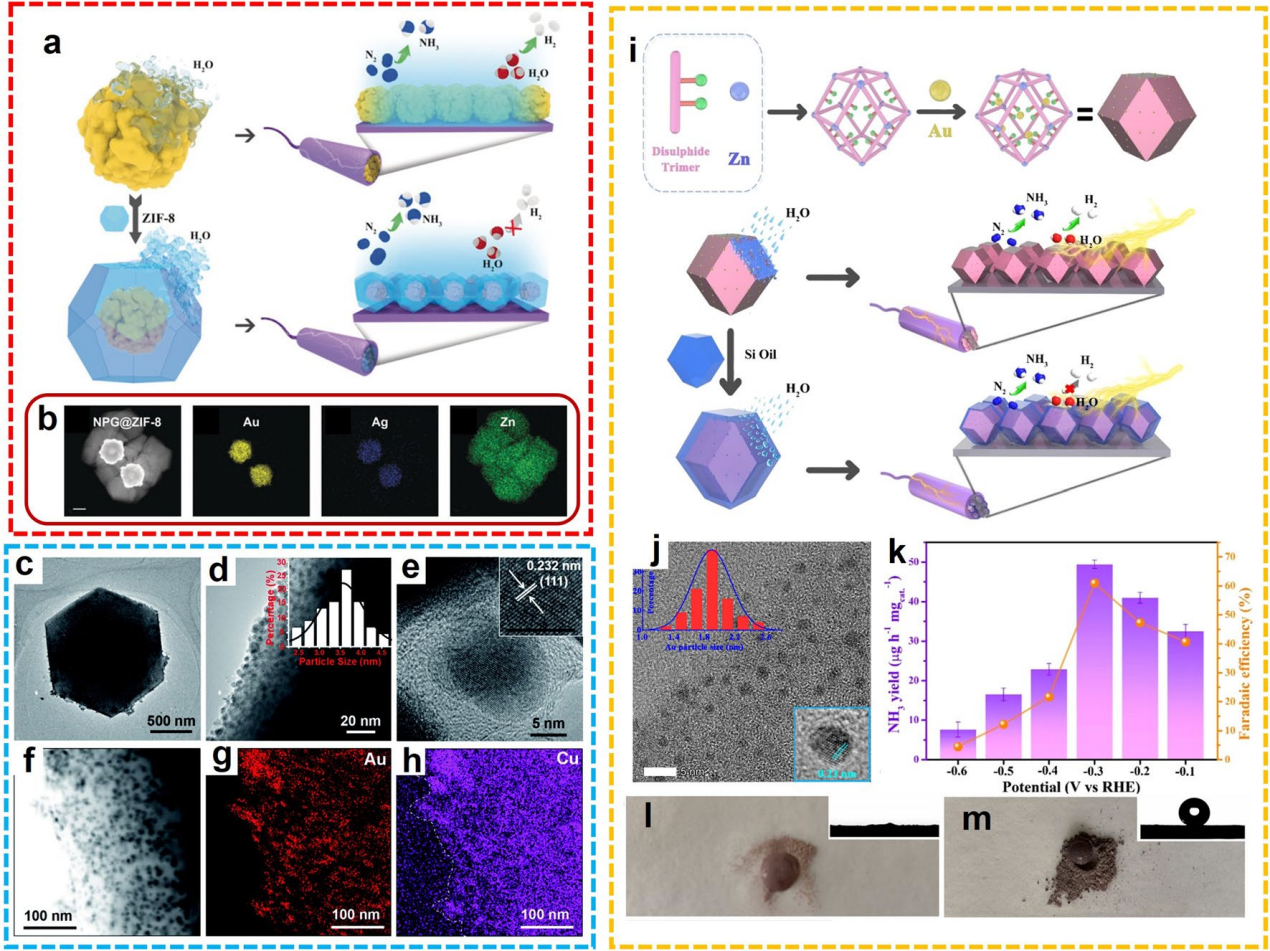
**a** Schematic diagram of nanoporous Au@ZIF-8 for NRR. **b** HAADF-STEM and EDS elemental mappings. Reproduced with permission from Ref. [64]. Copyright 2019, Wiley–VCH. **c**, **d** TEM images, **e** HRTEM image and **f** STEM image, and corresponding **g** Au and **h** Cu element mapping images of AuCu/ZIF-8 (Au/Cu raw molar ratio 3:7). Reproduced with permission from Ref. [65]. Copyright 2020, Royal Society of Chemistry. **i** Schematic diagram of HT Au@MOF composite for NRR. **j** HRTEM image. **k** Ammonia yield and FE at different potentials. Digital photos for **l** Au@MOF and **m** HT Au@MOF samples. Reproduced with permission from Ref. [66]. Copyright 2021, Elsevier

In the field of electrocatalyst development for the NRR process, noble metal nanoparticles have already demonstrated their high efficiency in the adsorption and activation of N_2_ molecules. However, the catalytic robustness always inclines to decrease dramatically attributed to the intrinsic weakness of the metal nanoparticles, which is prone to agglomeration as the reaction proceeds. Developing a proper strategy to well-disperse the metal nanoparticles and alleviate their tendency to agglomerate is an effective way for promoting catalytic efficiency. With rigid skeleton and well-defined pores, MOFs are a suitable substrate for catalytic active metal nanoparticles. Encapsulated in the channels or cavities of MOF structure, the metal nanoparticles are confined against the gathering. Moreover, the residue pores of the composites facilitate the mass transfer, while the competing HER process was restrained by the native hydrophobicity of MOF. Therefore, impregnation with metal nanoparticles can improve the catalytic performance of MOFs in the electrochemical N_2_ fixation process. For example, Yang et al. designed a composite of nanoporous gold and ZIF-8 (NPG@ZIF-8) with a core–shell structure (Fig. 7a, b) [64]. As the hydrophobicity of ZIF-8 reduces the proximity of H_2_O molecules to the catalyst, the ammonia yield and FE of NPG@ZIF-8 are significantly increased. At the same time, the micropores inherent in the ZIF structure can adsorb nitrogen on the surface of the catalyst. The core–shell structured metal nanoparticle@MOF composites are promising catalysts for the electrosynthesis of NH_3_ under ambient conditions. Furthermore, Lv et al. [65] prepared an Au-Cu alloy nanoparticle that could also facilitate electron transport between reactants and catalyst surfaces by introducing ZIF-8 to improve the limited specific surface area and exposed active sites (Fig. 7c-h). This enabled the catalyst to achieve an unprecedented ammonia yield of 23.3 µg h^−1^ mg_cat_.^−1^ with nitrogen and air as feedstocks and acidic electrolytic solutions in all pH ranges. Moreover, He and co-workers developed a hydrophobic Au@MOF coated with organosilicon (HT-Au@MOF) for NH_3_ generation [66]. The Au nanoparticles were enveloped in a thiol-equipped ZIF-8 skeleton through coordination interaction (Fig. 7i, j). In 0.1 M Na_2_SO_4_, the highest NH_3_ yield rate of 49.5 µg h^−1^ mg_cat._^−1^ and FE of 60.9% were realized at − 0.3 V versus RHE (Fig. 7k). In the NRR process, the HER was significantly suppressed by the hydrophobicity from the organosilicon layer, and outstanding catalytic properties were achieved attributed to the active Au catalytic site as well as the N_2_ aggregation effect of the porous ZIF-8 (Fig. 7l, m). More recently, Wen and co-workers fabricated a UiO-66-based composite with electron-rich PdCu nanoparticles implanted into the cavity and hydrophobic polydimethylsiloxane coating (PdCu@UiO-S@PDMS) [67]. The as-prepared MOF-hybrid was adopted as an efficient NRR electrocatalyst for the generation of NH_3_. Due to the integration of hydrophobicity, proton supply, and catalytic activity from each component of the PdCu@UiO-S@PDMS, the NH_3_ was successively produced with 20.24 µg h^−1^ mg_cat._^−1^ yield rate and 13.16% FE at − 0.25 V versus RHE in 0.1 M HCl.

**Fig. 8:**
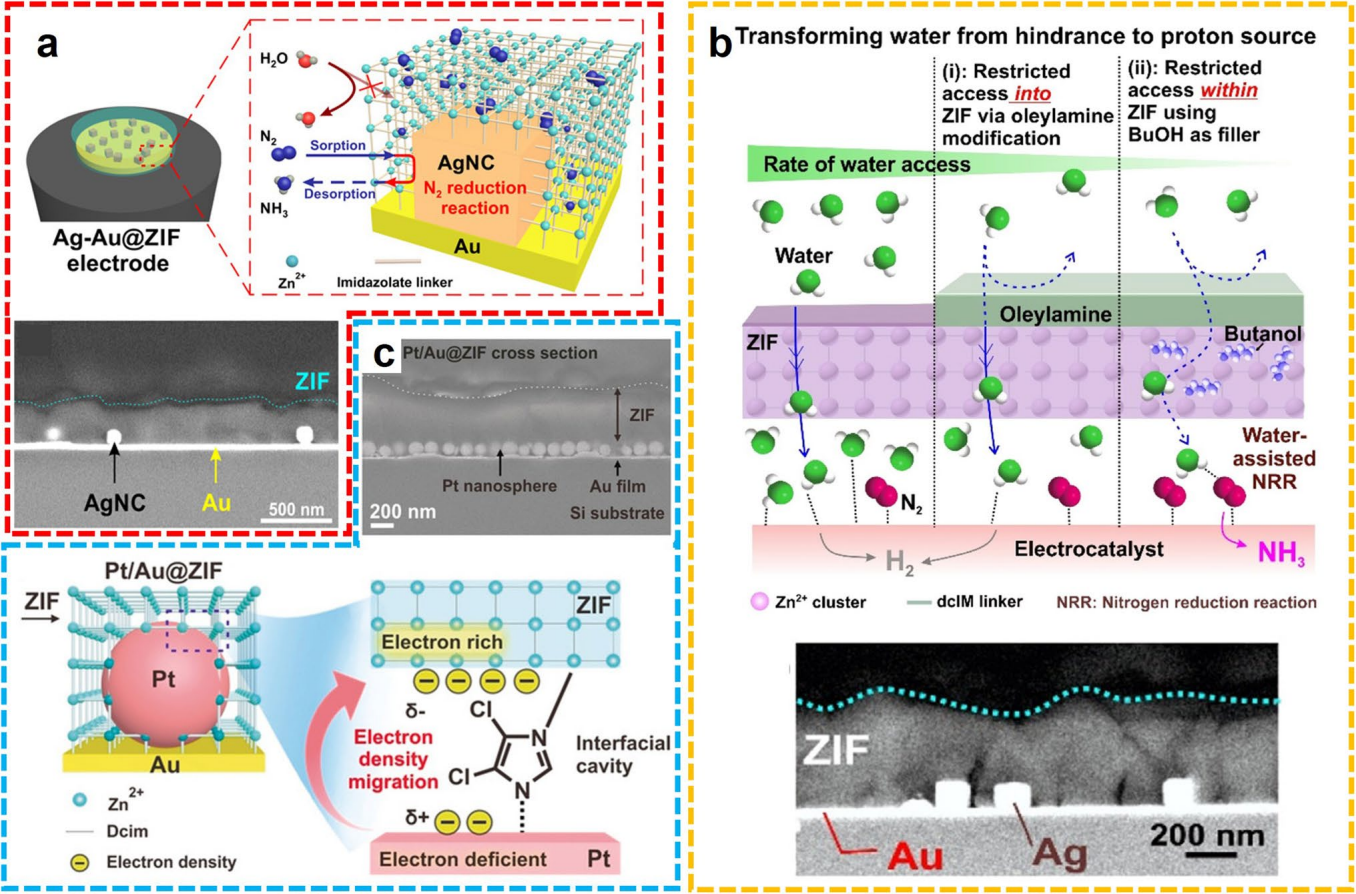
**a** Schematic diagram and the cross-sectional SEM image of Ag-Au@ZIF. Reproduced with permission from Ref. [68]. Copyright 2018, Science. **b** Schematic diagram and the cross-sectional SEM image of M@ZIF-OAm. Reproduced with permission from Ref. [70]. Copyright 2020, American Chemical Society. **c** Schematic diagram and the cross-sectional SEM image of Pt/Au@ZIF. Reproduced with permission from Ref. [69]. Copyright 2020, Wiley–VCH

In addition to the aqueous electrolytic solutions, the NRR conducted in organic solutions to bypass HER also exhibited superior performance with heterogeneous catalysts. For instance, Lee and colleagues deposited ZIF-71 on an Ag-Au platform using a wet chemical deposition method, fabricating a ZIF-coated Ag-Au electrode (Ag-Au@ZIF) for NH_3_ electrosynthesis in dry THF solution with LiCF_3_SO_3_ as the electrolyte (Fig. 8a) [68]. The significant suppression of competing water electrolysis was realized attributed to the water-repelling effect of the ZIF layer, and the enhanced catalytic performance was achieved due to the porous structure of ZIF that increases the concentration of nitrogen gas and prevents agglomeration of Ag-Au nanoparticles. Continuously, two similar works were proposed by the same group. For the electrocatalytic fixation of N_2_ to NH_3_ in THF media, a ZIF-encapsulated Pt/Au electrocatalyst has been synthesized and the d-band electronic structure of the bimetallic alloy has been modified, reducing the surface adsorption of a hydrogen atom to improve the NRR performance (Fig. 8b) [69]. In dry THF media, the N_2_ to NH_3_ process catalyzed by Pt/Au@ZIF achieves up to 4 times more FE than bare Pt/Au, obtaining a high FE of > 44% and an NH_3_ yield rate of > 161 µg h^−1^ mg_cat._^−1^ under ambient conditions. After experimental comparison and theoretical calculation, the originally unfavored N_2_ reduction process becomes favored due to the hydrophobic surface, concentrating effect, and electronic modification caused by the ZIF deposit. In another example, the authors confined metallic electrocatalysts in the ZIF-71 structure, fabricating the M@ZIF platform for high-selective NH_3_ production (Fig. 8c) [70]. Then, the hydrophobic functionalized oleyamine was installed superficially for water exclusion, and butanol was impregnated into the ZIF structure as a proton supplier. The performance of electrocatalytic N_2_ fixation was examined in a dry THF electrocatalytic solution. Notably, different from the other two works, the catalytic efficiency can be affected by the water content, which gradually increases in the interval 0–0.1% v/v and then decreases with the further growth of water. These works enriched the design and synthesis of proper electrocatalysts as well as the choice of electrolyte solutions for electrocatalytic NH_3_ synthesis.

**Fig. 9:**
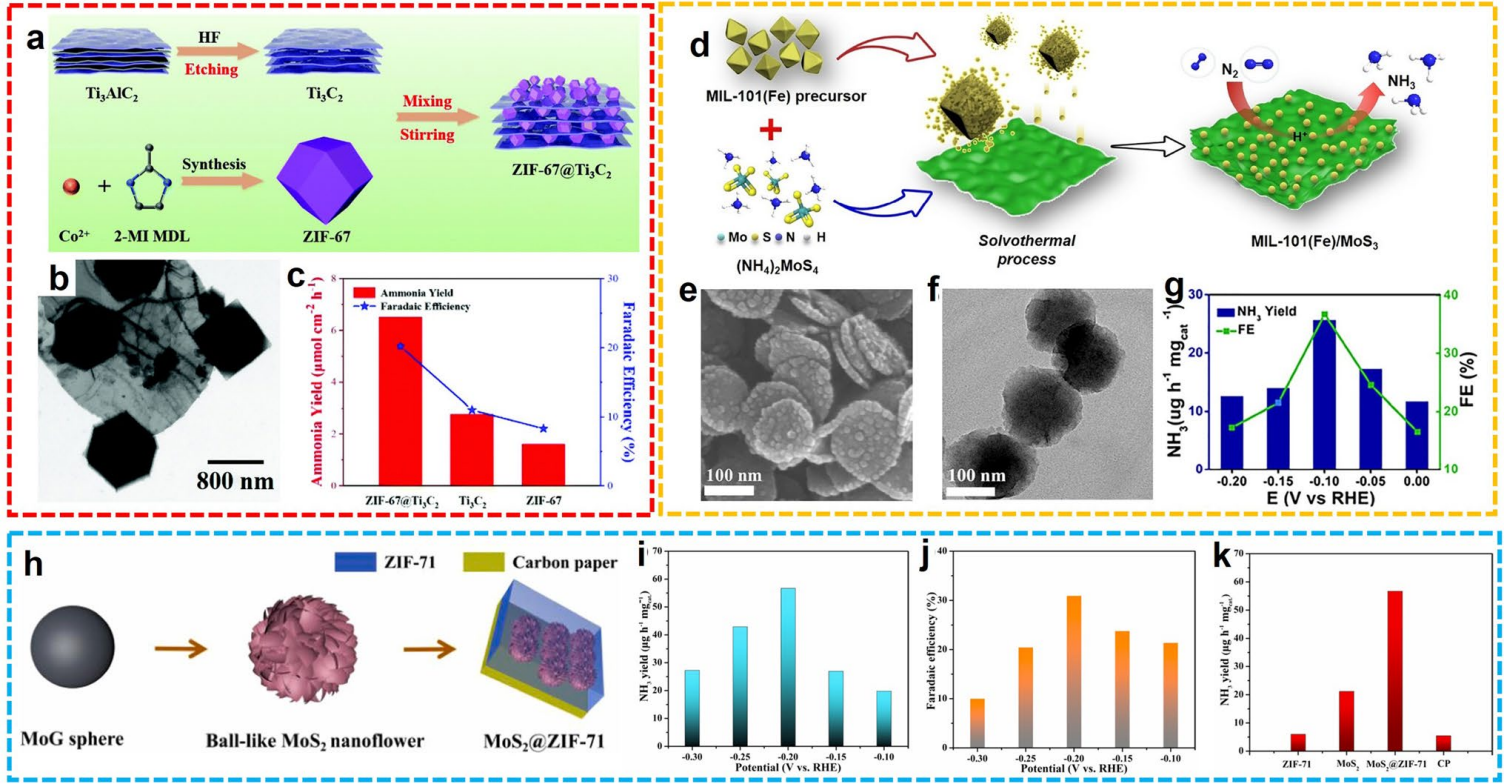
**a** Synthesis process and **b** TEM image of ZIF-67@Ti_3_C_2_. **c** NH_3_ yields and FEs of ZIF-67, Ti_3_C_2_, and ZIF-67@Ti_3_C_2_. Reproduced with permission from Ref. [72]. Copyright 2021, Royal Society of Chemistry. **d** Schematic diagram, **e** SEM image, **f** TEM image, and **g** NRR performance of MIL-101(Fe)/MoS_3_. Reproduced with permission from Ref. [73]. Copyright 2022, Springer Nature. **h** Synthesis process and **i**-**k** NRR performance of MoS_2_@ZIF-71. Reproduced with permission from Ref. [74]. Copyright 2021, Elsevier

Recently, thin-layered 2D nanomaterials exhibited their superior catalytic performance in the electrochemical conversion of simple molecules [71]. After the hybridization with MOFs, these nanocomposites can elevate the product selectivity and further enhance the catalytic efficiency. For example, Liang et al. prepared ZIF-67 on the Ti_3_C_2_ layer by in situ growth method for NH_3_ synthesis (Fig. 9a, b) [72]. By virtue of the high porosity and large specific surface area of ZIF as well as the excellent electrical conductivity of 2D Ti_3_C_2_, the composite material ZIF-67@Ti_3_C_2_ exhibited excellent NH_3_ yield of 6.52 μmol h^−1^ cm^−2^ and FE of 20.2% at − 0.4 V versus RHE in 0.1 M KOH. Notably, due to the synergistic effect of ZIF-67 and Ti_3_C_2_, the electrocatalytic activity of composite ZIF-67@Ti_3_C_2_ for N_2_ fixation is significantly higher than individual components, ZIF-67 and Ti_3_C_2_ (Fig. 9c). In another work, Xu et al. developed a MIL-101(Fe) modified MoS_3_ nanocomposite (MIL-101(Fe)/MoS_3_) for artificial N_2_ fixation (Fig. 9d) [73]. The hybrid material possesses a homogeneous nanolayer of MoS_3_ and crystal nanodots of MIL-101(Fe) (Fig. 9e, f). In 0.1 M HCl, the NH_3_ transformation from N_2_ was achieved with 25.7 µg h^−1^ mg_cat._^−1^ yield rate and 36.71% FE at − 0.1 V versus RHE (Fig. 9g). Similarly, Duan and co-workers developed ZIF-71 enveloped ball-like MoS_2_ nanoflowers (MoS_2_@ZIF-71) via layer-by-layer growth strategy for electrocatalytic NRR application (Fig. 9h) [74]. Catalyzed by MoS_2_/CP electrode coated with ZIF-71, the NH_3_ products were provided in a maximum of 56.69 µg. h^−1^ mg_cat._^−1^ yield rate and 30.91% FE at − 0.2 V versus RHE in 0.1 M Na_2_SO_4_ (Fig. 9i, j). The hydrophobicity of porous ZIF-71 is responsible to concentrate N_2_ and suppress HER, and the synergistic effect between MoS_2_ and ZIF-71 was intensively confirmed by the control experiments (Fig. 9k). These aforementioned advances put forward innovative strategies for fabricating hybrid composites of 2D nanostructure and MOF, and expanded the choices of promising candidates of electrocatalysts for the NRR process.

Furthermore, the carbon-based catalysts containing defects present a positive effect in adsorbing and activating N_2_ molecules, thus improving the process of N_2_ fixation. The integration of conductivity and catalytic activity of carbon nanotubes (CNTs) and N_2_ enrichment and hydrophobicity of MOFs make the CNT@MOF composites promising NRR electrocatalysts for practical use. Lv et al. proposed a combined catalytic method of MOF doping CNT or N-doped CNT (NCNT) for electrochemical N_2_ fixation [75]. The MOF dopant enlarged the water contact angle and N_2_ sorption uptake of the material, inferring improved water-repelling ability for rationally restraining HER and N_2_ enrichment for promoted N_2_ fixation.

Based on the aforementioned works listed in Table 1, pristine MOFs and MOF-hybrid materials are certificated as promising electrocatalysts for efficient artificial N_2_ fixation for NH_3_. In the framework, the metals are generally considered to have the ability to adsorb and activate N_2_ molecules, while hydrophobic organic linkages are supposed to suppress the competing HER process. Meanwhile, the porous skeletons not only provide sufficient space to accelerate the reduction of N_2_ but also offer the opportunity to couple with other functional materials, which facilitates the integration and optimization for better catalytic performance. Additionally, conductive MOFs and 2D MOF nanosheets exhibit their unique promotion effect for electrocatalytic N_2_-to-NH_3_, such as enhanced conductivity, more exposed active sites, and more rapid mass transfer, etc.

**Table 1:** The performance of different MOFs and their related products in NRR

Catalyst	Electrolytic solution	Ammonia yield	FE	Potential	Refs.
Fe-MOF	2 M KOH	7.63 × 10^−3^ mmol h^−1^ cm^−2^	1.43%	1.2 V versus RHE	[51]
NH_2_-MIL-88B-Fe	0.1 M Na_2_SO_4_	1.205 × 10^–10^ mol s^−1^ cm^−2^	12.45%	0.05 V versus RHE	[52]
HKUST-1	0.1 M Na_2_SO_4_	46.63 μg h^−1^ mg_cat._^−1^	2.45%	− 0.75 V versus RHE	[53]
JUC-1000/CC	1 M Na_2_SO_4_	24.7 μg h^−1^ mg_cat_.^−1^	11.9%	0.4 V versus RHE	[54]
Co_3_(HHTP)_2_	0.5 M LiClO_4_	22.14 μg h^−1^ mg_cat._^−1^	3.34%	− 0.4 V versus RHE	[55]
MIL-100-Al	0.1 M KOH	10.6 μg h^−1^ cm^−2^	22.6%	0.05 V versus RHE	[56]
Defective UiO-66-NH_2_	0.1 Na_2_SO_4_	52.81 μg h^−1^ mg_cat_.^−1^	85.21%	− 0.39 V versus RHE	[57]
Cu@Ce-MOF	0.1 M KOH	14.83 μg h^−1^ cm^−2^	10.81%	− 0.2 V versus RHE	[58]
In-MOF	0.01 M KOH (pH = 12)	79.20 μg h^−1^ mg_cat._^−1^	14.98%	− (0.5 mA cm^−2^)	[59]
Fe-TCPP	0.1 HCl	44.77 μg h^−1^ mg_cat._^−1^	16.23%	− 0.3 V versus RHE	[60]
OPA-PCN-222(Fe)	0.1 HCl	49.7 μg h^−1^ mg_cat_.^−1^	17.2%	− 0.5 V versus RHE	[61]
NiFe-MOF	0.1 M NaHCO_3_	9.3 μg h^−1^ mg_cat._^−1^	11.5%	− 0.345 V versus RHE	[62]
Co_3_Fe-MOF	0.1 M KOH	8.79 μg h^−1^ mg_cat_.^−1^	25.64%	− 0.2 V versus RHE	[63]
NPG@ZIF-8	0.1 M Na_2_SO_4_	28.7 μg h^−1^ cm^−2^ (− 0.8 V)	44% (− 0.6 V)	− versus RHE	[64]
AuCu/ZIF-8	0.1 M HCl	63.9 μg h^−1^ mg_cat._^−1^ (− 0.2 V)	14.2% (0 V)	− versus RHE	[65]
HT Au@MOF	0.1 M Na_2_SO_4_	49.5 μg h^−1^ mg_cat_.^−1^	60.9%	− 0.3 V versus RHE	[66]
PdCu@UiO-S@PDMS	0.1 HCl	20.24 μg h^−1^ mg_cat_.^−1^	13.16%	− 0.25 V versus RHE	[67]
Ag-Au@ZIF	0.2 M LiCF_3_SO_3_ (≈1% ethanol + THF)	0.623 μg h^−1^ cm^−2^	18%	− 2.9 V vs Ag/AgCl	[68]
M@ZIF-OAm	0.2 M LiCF_3_SO_3_ (≈1% butanol + 0.1% H_2_O + THF)	48.2 μg h^−1^ cm^−2^	19%	− 0.29 V versus RHE	[69]
Pt/Au@ZIF	0.2 M LiCF_3_SO_3_ (≈1% ethanol + THF)	161 μg h^−1^ mg_cat._^−1^	44%	− 2.9 V vs Ag/AgCl	[70]
ZIF-67@Ti_3_C_2_	0.1 M KOH	6.52 μmol h^−1^ cm^−2^	20.2%	− 0.4 V versus RHE	[72]
MIL-101(Fe)/MoS_2_	0.1 HCl	25.7 μg h^−1^ mg_cat_.^−1^	36.71%	− 0.1 V versus RHE	[73]
MoS_2_@ZIF-71	0.1 M Na_2_SO_4_	56.69 μg h^−1^ mg_cat._^−1^	30.91%	− 0.2 V versus RHE	[74]
CNT@MIL-101(Fe)	0.05 H_2_SO_4_	5.514 μg h^−1^ mg_cat._^−1^	37.28%	− 0.45 V versus RHE	[75]

### MOF-derived Electrocatalysts

MOFs can also be used as pre-catalysts. Through proper pyrolysis or calcination processes, the coordinatively fabricated MOF can be derived into carbon-based nanomaterials with enhanced conductivity and stability, thus further improving the electrocatalytic efficiency. In most cases, the framework can be reserved or slightly shrank with organic linkage decomposed into carbon materials, while the metal residues were converted into metal oxides, metal sulfides, metal carbides, phosphating compounds, selenides, and even single metal atoms as catalytic active sites that were firmly installed on the carbon-based materials. The following contents are mainly focused on the MOF-derived electrocatalysts for NRR, such as porous carbon catalysts, single-atom catalysts, and other nanostructured composites.

#### Heteroatom-doped Porous Carbon Catalysts

**Fig. 10:**
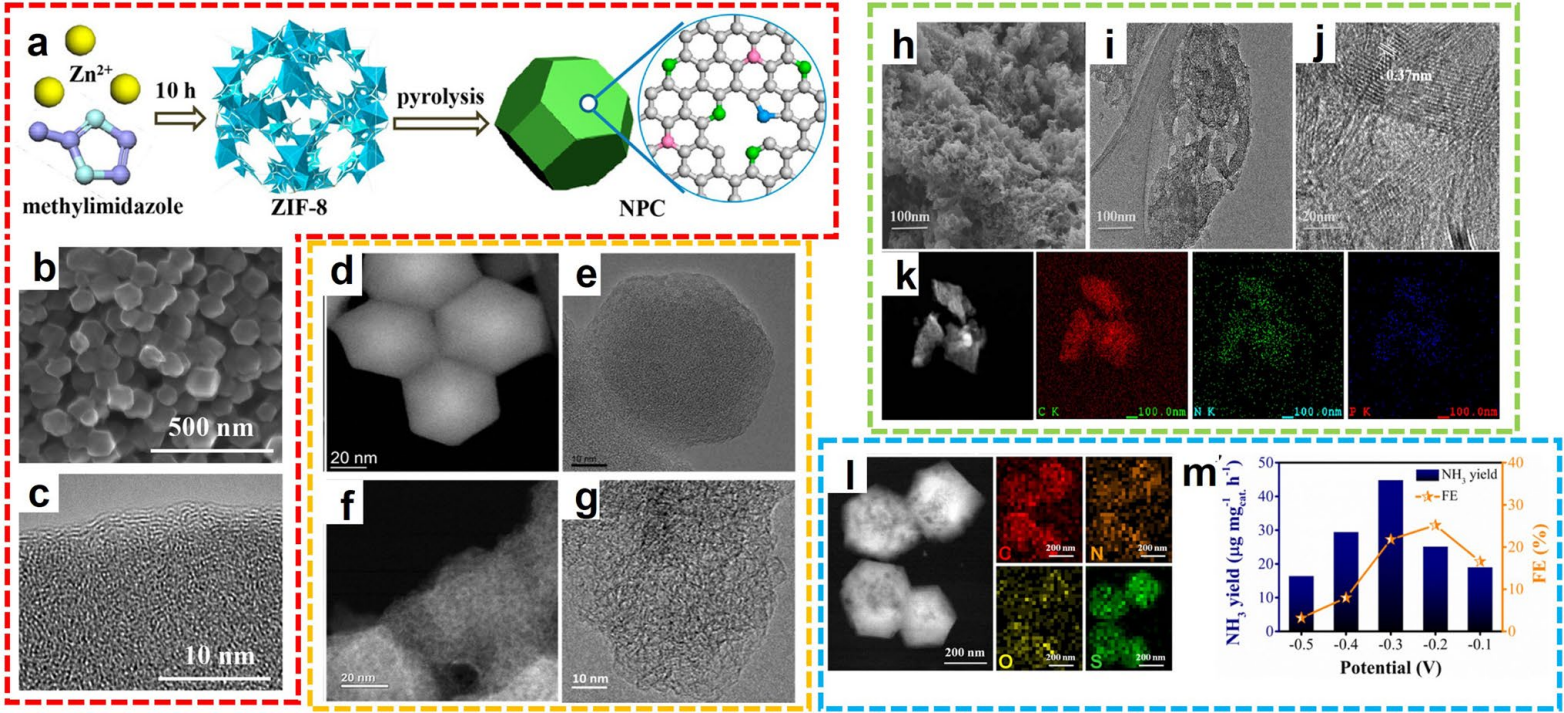
**a** Schematic illustration of NPC preparation. **b** SEM image and **c** SEM image of NPC-750. Reproduced with permission from Ref. [77]. Copyright 2018, American Chemical Society. Comparison of morphology and microstructure between **d**, **e** ZIF-8 nanocrystal precursors and **f**, **g** pyrolyzed C-ZIF-1100–1. Reproduced with permission from Ref. [78]. Copyright 2018, Elsevier. **h** SEM image, **i**, **j** TEM images, and **k** elements mappings of NP-C-MOF-5. Reproduced with permission from Ref. [79]. Copyright 2019, American Chemical Society. **l** Elements mappings and **m** NRR performance of S/N-MPC. Reproduced with permission from Ref. [80]. Copyright 2021, Elsevier

By virtue of abundant origins, fabricable structure, and tunable function, non-metal catalysts have become more and more attractive in various electrochemical transformations [76]. Among the reported non-metal catalysts, MOF-derived carbons received much attention due to their merits of high surface area, adjustable porosity, and excellent thermal stability. Most importantly, changing the pyrolysis temperature can adjust the degree of graphitization, which can change the electronic and geometric structure of carbon, and thus improve the performance of NRR. For example, Liu et al. prepared a cost-effective N-doped porous carbon (NPC) by carbonized ZIF-8 and utilized it as electrocatalysts for NH_3_ synthesis (Fig. 10a-c) [77]. The N_2_ adsorption was facilitated by the nitrogen content, and pyridinic and pyrrolic nitrogen were verified as active sites for the fixation of N_2_. Compared with transition metals, metal-free MOF-derived N-doped carbon significantly inhibited HER activity in the acidic media (0.05 M H_2_SO_4_), thus promoting the NH_3_ yield reached 1.40 mmol g^−1^ h^−1^ at − 0.9 V versus RHE. Moreover, similar work was also reported by Mukherjee and colleagues that the ZIF-derived carbon catalyst exhibited encouraging activity and stability for electrochemical N_2_ conversion in alkaline media (Fig. 10d-g) [78]. The C-ZIF-1100-1 was prepared from the pyrolysis of ZIF-8 precursor at 1100 °C for 1 h, providing the NH_3_ with FE up to 10.2% at − 0.3 V versus RHE 0.1 M KOH. Intriguingly, the N_3_ site of N-doped carbon formed during high-temperature pyrolysis was claimed to be the catalytic active center for the electrochemical reduction of N_2_. Both examples indicated the ZIF-derived NPC being a low-cost metal-free catalyst for NH_3_ generation via N_2_ reduction.

In addition to the N-dopants, the NPC materials can be further activated by the introduction of other heteroatoms, providing more active sites for adsorption and reduction of N_2_ molecules. For example, Song et al. prepared N, P co-doped porous carbon electrocatalysts from MOF precursors [79]. Through the carbonization of MOF-5, dicyandiamide, and triphenylphosphine mixture at 900 °C, NP-C-MOF-5 was fabricated and adopted as the electrocatalyst for electrocatalytic N_2_ fixation (Fig. 10h-k). In acidic electrolytic solutions, the NH_3_ was obtained with the yields of 1.08 µg h^−1^ mg_cat._^−1^. As another example, Wang et al. prepared sulfur-modified N-MPC from metal azolate frameworks (MAFs) for electrocatalysis of N_2_ conversion [80]. The morphology and structure of MAF were maintained in the pyrolyzed N-MPC, and the introduction of sulfur provided an active site for N_2_ adsorption and subsequent reduction (Fig. 10l). Experimental results show that S/N-MPC exhibited excellent N_2_-to-NH_3_ selectivity and electrochemical stability, offering the NH_3_ yield of 45.51 μg h^−1^ mg_cat_.^−1^ and FE of 25.16% at − 0.3 and − 0.2 V versus RHE, respectively (Fig. 10m). Both examples demonstrated that the incorporation of heteroatoms into carbon-based electrocatalysts can improve NRR performance.

#### Single-atom Catalysts

Single-atom catalysts (SACs) are widely used in various electrocatalytic reduction processes because of their unique electronic structure, uniform low coordination environment, atomically dispersed active sites, and maximum atomic utilization [28]. In general, MOF-derived SAC catalysts were constructed through the pyrolysis or carbonization of MOF precursors with metal embedded or installed. The highly dispersive metal atoms can be stabilized by the defect-rich supporting carbon substrates. With the merits of electric conductivity, well-defined porous structure, and quasi-molecular catalytic activity, the as-prepared SAC catalysts presented enhanced performance of electrochemical NRR.

**Fig. 11:**
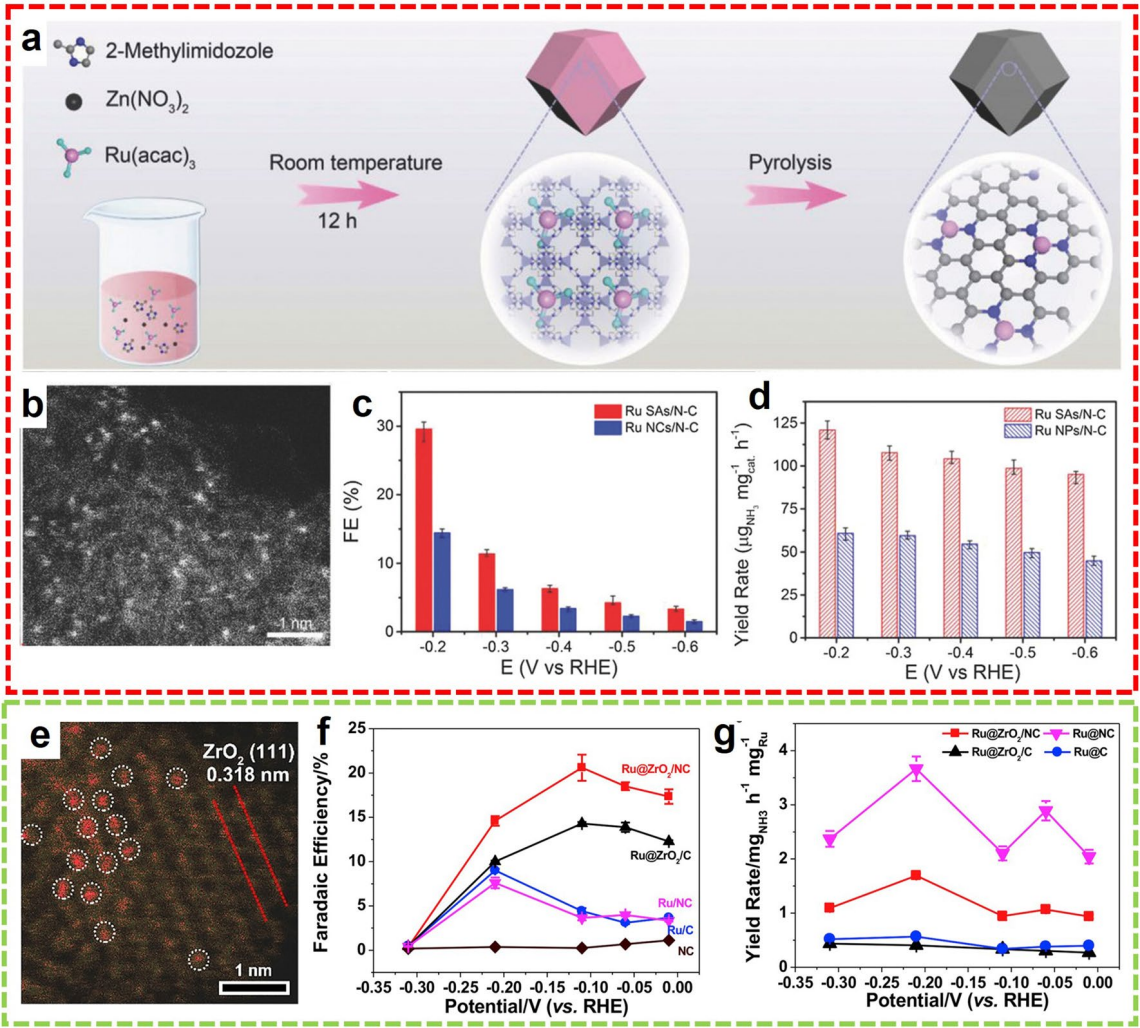
**a** Synthetic procedure and **b** HAADF-STEM image of Ru SAs/N–C. **c** FEs and **d** NH_3_ yields of Ru SAs/N–C and Ru NCs/N–C. Reproduced with permission from Ref. [81]. Copyright 2018, WILEY. **e** HAADF-STEM image of Ru@ZrO_2_/NC. **f** FEs and **g** NH_3_ yields of Ru@NC, Ru@C, Ru@ZrO_2_/NC, and Ru@ZrO_2_/C. Reproduced with permission from Ref. [82]. Copyright 2019, Elsevier

Among the many precious metal-based SACs, Ru-based SAC is located at the top of the volcano map, providing more opportunities for NRR. Geng et al. synthesized the nitrogen-doped carbon with dispersive Ru single atoms (Ru SAs/N–C) via pyrolysis of ZIF-8 with Ru-dopants and used them as electrocatalysts for the NRR process (Fig. 11a, b) [81]. At − 0.2 V versus RHE, the NH_3_ was produced with an FE of 29.6% and a record-breaking yield of 120.9 µg h^−1^ mg_cat._^−1^, still the top-ranked NH_3_ yield so far (Fig. 11c,d). Furthermore, Tao et al. developed a ZrO_2_/N-doped carbon composite catalyst with single Ru sites (Ru@ZrO_2_/NC) for the electrosynthesis of NH_3_ from N_2_ [82]. Ru ions were encapsulated in UiO-66 by a hydrothermal method and then annealed to generate the Ru@ZrO_2_/NC in N_2_. HAADF-STEM observations confirmed the distribution of single Ru sites on the carbon support, the size of most of the Ru atoms fell in the range of 0.1–0.2 nm (Fig. 11e). The formation of atom-dispersed Ru is most likely due to uncoordinated -NH_2_ groups stabilizing the precursor and inhibiting Ru aggregation during pyrolysis. At a more negative than − 0.31 V versus RHE, the FE of NH_3_ approaches 0 due to competitive HER for Ru@NC, whereas the addition of ZrO_2_ significantly promoted the generation of NH_3_ at all applied potentials. Catalyzed by the Ru@ZrO_2_/NC, the FE of NH_3_ reaches a maximum of about 21% at − 0.11 V versus RHE and the NH_3_ yield of 3.665 mg h^−1^mg_cat._^−1^ at − 0.21 V versus RHE (Fig. 11f, g).

**Fig. 12:**
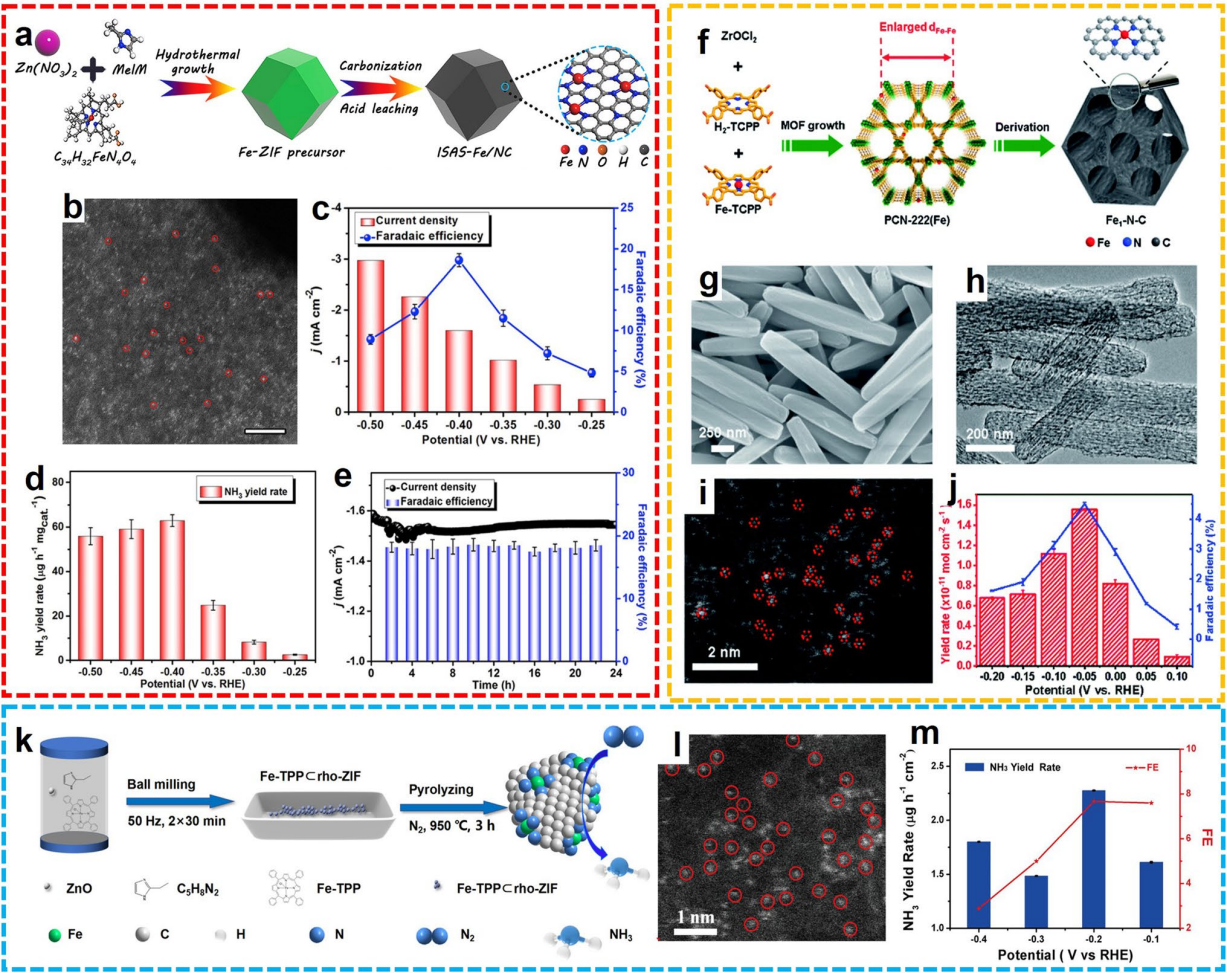
**a** Schematic synthesis route, **b** HAADF-STEM image, **c**-**e** electrochemical NRR performance of ISAS-Fe/NC. Reproduced with permission from Ref. [83]. Copyright 2019, Elsevier. **f** Schematic synthesis route of Fe_1_-N–C. TEM image of **g** PCN-222(Fe) precursors and **h** Fe_1_-N–C. **i** HAADF-STEM image and **j** NRR performance of Fe_1_-N–C. Reproduced with permission from Ref. [84]. Copyright 2019, Royal Society of Chemistry. **k** Schematic illustration, **l** HAADF-STEM image, and **m** NRR performance of Fe–N/C-CPs. Reproduced with permission from Ref. [85]. Copyright 2021, American Chemical Society

In addition to Ru SACs, the transition-metal SACs were also demonstrated as effective catalysts for the electrochemical artificial fixation of N_2_. For example, Lü et al. fabricated N-doped carbon frameworks anchored with isolated Fe single atoms (ISAS-Fe/NC) through carbonization and etching of pre-synthesized bimetallic Fe-doped ZIF-8 (Fig. 12a, b) [83]. The as-prepared Fe SAC exhibited superior catalytic performance for the generation of NH_3_, with the NH_3_ yield of 62.9 ± 2.7 μg h^−1^ mg_cat_.^−1^ and FE of 18.6 ± 0.8% at − 0.4 V versus RHE in neutral media (Fig. 12c, d). The electrocatalytic stability of ISAS-Fe/NC was verified by a long-term electrolysis test, with slight fluctuation of current density in 24 h (Fig. 12e). This work shed light on the practical use of low-cost transition-metal SACs in environmentally friendly NH_3_ synthesis with low energy consumption. Furthermore, the Fe SACs were also developed from the pyrolysis of PCN-222(Fe) precursors (Fe_1_–N–C), reported by Zhang and co-workers (Fig. 12f) [84]. The nanorod shape of Fe_1_–N–C was inherited from the MOF precursor, and the atomically dispersed Fe sites were confirmed by the HAADF-STEM image (Fig. 12g-i). At a relatively low overpotential, − 0.05 V versus RHE, the maximum NH_3_ yield reached 1.56 × 10^–11^ mol cm^−2^ s^−1^ with 4.51% FE (Fig. 12j). In another example, Liu et al. prepared a Fe–N/C SAC from the pyrolysis of Fe-TPP/ZIF-8 precursors (Fig. 12k, l) [85]. Obtained through ball milling, the precursors were annealed and subsequently fixed on pre-treated carbon papers by Nafion. Then, the electrosynthesis of NH_3_ was mediated by the as-fabricated Fe–N/C-CP catalytic electrode, realizing the NH_3_ yield of 2.27 μg h^−1^ mg_cat_.^−1^ with 7.67% FE at − 0.2 V versus RHE (Fig. 12m).

**Fig. 13:**
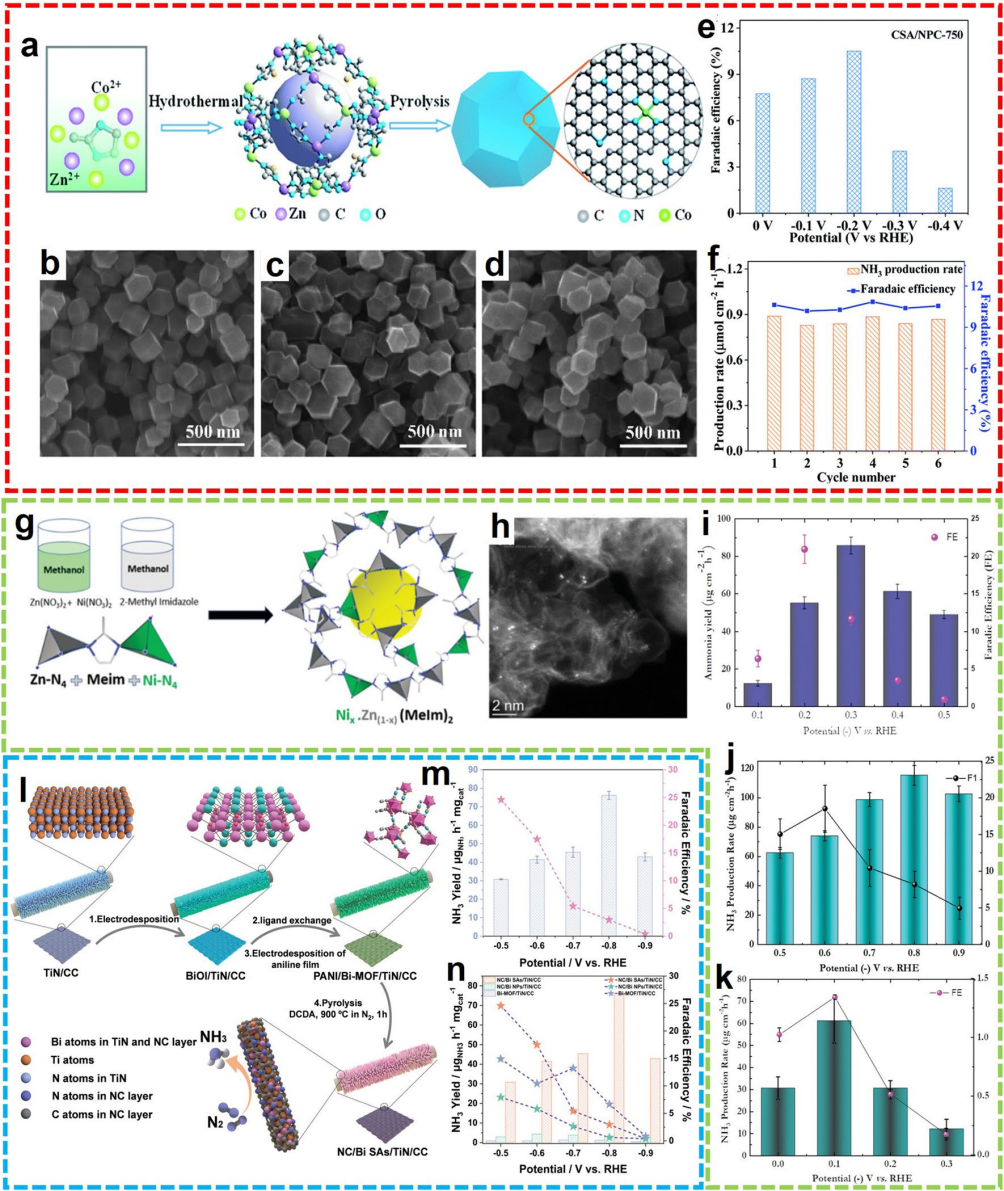
**a** Schematic synthesis route and **b**-**c** TEM images with different annealing temperatures of CSA/NPC. **e** FE at different potentials and **f** duration test of CSA/NPC-750. Reproduced with permission from Ref. [86]. Copyright 2019, Royal Society of Chemistry. **g** Schematic of Ni_*x*_Zn_(1-*x*)_BMOF. **h** HAADF-STEM image of Ni-N_*x*_-C. NRR performance of Ni-N_*x*_-C-700-3h **i** in alkaline media, **j** in neutral media, and **k** in acidic media. Reproduced with permission from Ref. [88]. Copyright 2020, WILEY. **l** Schematic illustration and **m** NRR performance of NC/Bi SAs/TiN/CC. **n** NRR performance comparison with related catalysts. Reproduced with permission from Ref. [89]. Copyright 2022, WILEY

Apart from Fe, advances using other transition-metal-based SACs were also proposed for the investigation of electrocatalysts in NRR. For instance, Liu et al. prepared Co- single atom embedded N-doped porous carbon (CSA/NPC) by annealing the Co-doped ZIF-8 (Fig. 13a) [86]. The polyhedral crystal morphology of ZIF can also be observed in the CSA/NPC (Fig. 13b-d). And the NH_3_ yield and FE were 0.86 µmol cm^−2^ h^−1^ and 10.5%, respectively (Fig. 13e). The as-prepared CSA/NPC not only has high NRR activity and selectivity but also has good electrochemical stability (Fig. 13f). Furthermore, Gao et al. synthesized a cobalt/nitrogen-doped porous carbon (Co/NC) electrocatalyst consisting of a single Co site by carbonizing ZIF-67. In 0.1 M KOH, the maximum NH_3_ yield was 5.1 μg h^−1^ mg_cat_.^−1^ at − 0.4 V versus RHE, and the FE reached up to 10.1% at − 0.1 V versus RHE [87]. In another case, Mukherjee and colleagues prepared an atomically dispersed Ni site electrocatalyst through the pyrolysis of the Ni-Zn bimetallic organic framework (Fig. 13g) [88]. With the partial replacement of Zn nodes by Ni during the formation process, the Ni-N_*x*_-C coordination structure was formed after annealing (Fig. 13h). Notably, the formation of NH_3_ was not limited by the pH of the electrolytic solution, yielding the optimized NH_3_ yield of 115 µg cm^−2^ h^−1^ at − 0.8 V versus RHE under neutral conditions, and a high FE of 22.9% at − 0.2 V versus RHE under alkaline conditions (Fig. 13i-k).

Compared with precious metals, low-cost semi-metals are also listed as candidate materials for NRR, especially nanoscale Bi. Bi-based NRR electrocatalysts with ideal electronic structure and high-exposure active sites can be prepared through reasonable composite design. Xi et al. prepared hollow titanium nitride nanorods bound by monoatomic Bi and fixed them in NC loaded on carbon cloth (NC/Bi SAs/TiN/CC) (Fig. 13l) [89]. The composite exhibits a superior NH_3_ yield rate of 76.15 μg h^−1^ mg_cat._^−1^ at − 0.8 V versus RHE and a high FE of 24.60% at − 0.5 V versus RHE in 0.1 M Na_2_SO_4_ solution (Fig. 13m). In addition, the synergistic effect of Bi-SAs and TiN can simultaneously promote N_2_ hydrogenation and inhibit HER (Fig. 13n).

Based on the above advances in electrochemical NRR promoted by the MOF-derived SACs, the precisely designed synthetic route of electrocatalysts play a vital role in enhancing energy conversion and NH_3_ generation. Thanks to the N-doped porous carbon substrates, the electric conductivity, proton transportation, and stabilization of single metal atoms were guaranteed, thus providing superior catalytic performance for artificial N_2_ fixation.

#### Other MOF-Derived Nanostructures

**Fig. 14:**
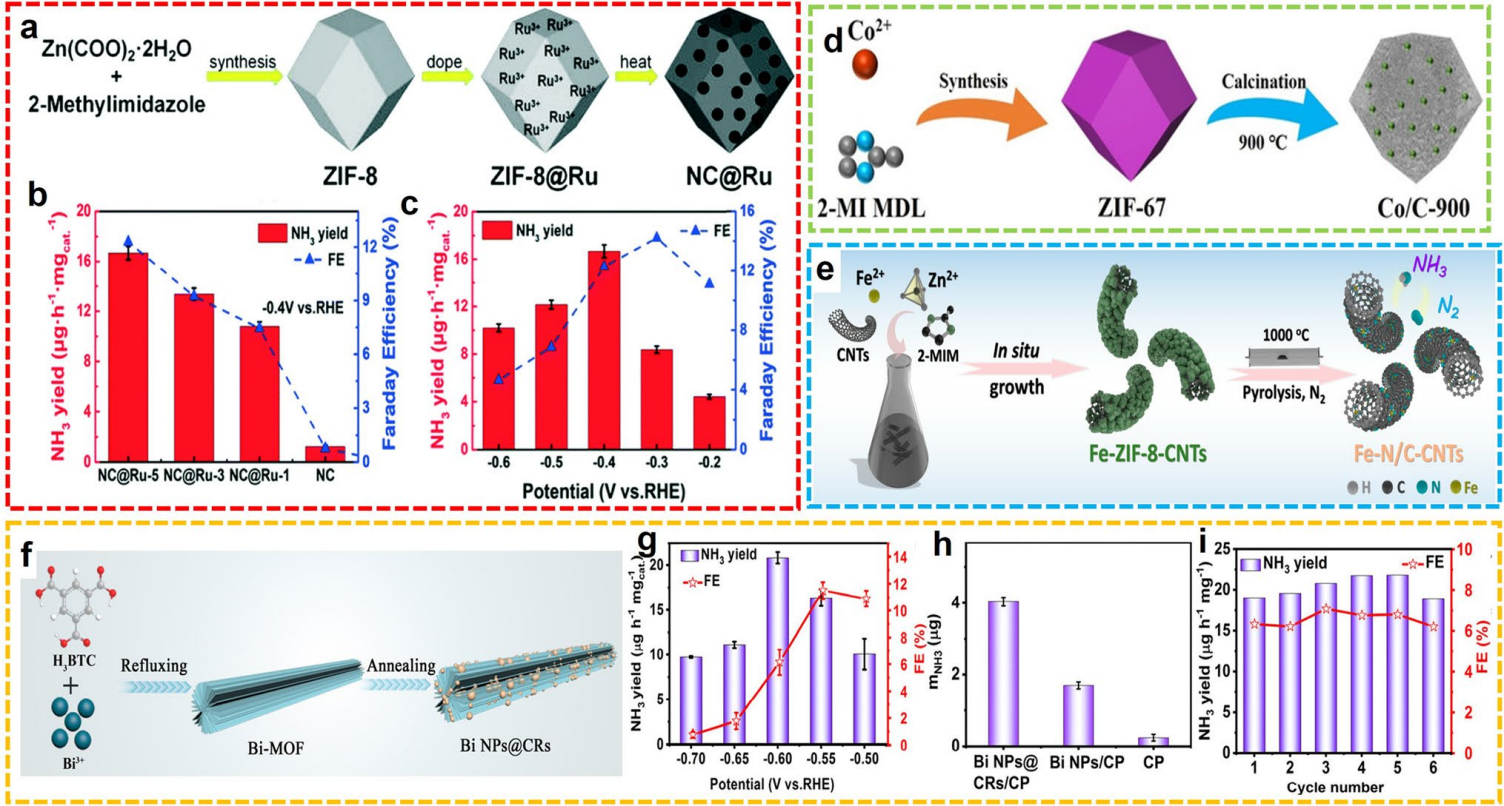
**a** Schematic synthesis route and **b**, **c** NRR performance of NC@Ru. Reproduced with permission from Ref. [90]. Copyright 2020, Royal Society of Chemistry. **d** Schematic synthesis of Co/C-900. Reproduced with permission from Ref. [91]. Copyright 2020, WILEY–VCH. **e** Schematic illustration of Fe–N/C-CNTs. Reproduced with permission from Ref. [93]. Copyright 2019, American Chemical Society. **f** Schematic synthesis and **g**-**i** NRR performances of Bi NPs@CRs. Reproduced with permission from Ref. [94]. Copyright 2021, American Chemical Society

Apart from the metal SACs, other MOF-derived nanostructures by high-temperature pyrolysis were also proved to be effective electrocatalysts in NRR for NH_3_ synthesis, including the carbon materials that dispersed by metal nanoparticles, metal oxides, metal sulfides, and metal phosphides. In general, the morphology of MOF precursors can be maintained after the pyrolysis, yielding porous carbon materials with dispersive metal sites. For example, Zhang et al. developed a rhombic shape Ru NPs dispersed N-doped carbon framework (NC@Ru) by carbonization of Ru-doped ZIF-8 at high temperatures (Fig. 14a) [90]. According to the comparison of NRR performance, NC@Ru-5 was verified to be one of the optimal catalyst, providing the highest yield of NH_3_ is 16.68 μg h^−1^mg_cat.._^−1^ at − 0.4 V versus RHE and that of FE is 14.23% at − 0.3 V vs RHE in 0.1 M KOH electrolytic solution (Fig. 14b, c). Meanwhile, without the introduction of external metal sources, the Co/C-900 composite was developed by Liu et al. through the direct calcination of ZIF-67 at 900 °C for 1 h in N_2_ (Fig. 14d) [91]. The Co nodes in ZIF-67 were fully adapted to the Co/C-900 as catalytic active sites. The N_2_ reduction was measured using Co/C-900 as the catalysts in 0.1 M KOH, displaying the highest FE of 11.53% and the maximum NH_3_ production rate of 4.66 μmol cm^−2^ h^−1^ at − 0.3 V versus RHE. Similarly, another MOF-derived Co-based nanomaterial was also proposed by Yin et al. [92]. The Co@N-doped carbon materials (Co@NC) were fabricated by ZIF-67 precursor annealing at high temperatures. Under ambient conditions, Co@NC exhibits excellent electrocatalytic performance. At − 0.9 V versus Ag/AgCl, the NH_3_ was offered an NH_3_ yield of 1.57 × 10^–10^ mol s^−1^ cm^−2^ with FE up to 21.79%. Furthermore, Wang et al. synthesized an electrocatalyst at the Fe-N3 site using Fe-ZIF-CNT composites as templates (Fig. 14e) [93]. In 0.1 M KOH media, this catalyst exhibits enhanced NRR activity with NH_3_ production of 34.83 μg h^−1^ mg_cat_.^−1^, and FE of 9.28% at − 0.2 V versus RHE. In 2021, Wang et al. developed an efficient electrocatalyst for the N_2_-to-NH_3_ process by confining Bi NPs into carbon rods (CRs) (Bi NPs@CRs) through annealing of Bi-MOF@CRs (Fig. 14f) [94]. In 0.1 M HCl, a high FE of 11.5% and a large NH_3_ yield of 20.8 μg h^−1^mg_cat._^−1^ for Bi NPs@CRs were obtained at − 0.55 V and − 0.6 V versus RHE, respectively (Fig. 14g). The superior catalytic activity was verified by the comparison with Bi NPs (Fig. 14h), and the stability was further evaluated through the test with 6 cycles (Fig. 14i). More recently, Wu et al. developed an N, P co-doped carbon catalyst with Bi anchored (Bi/NPC) for electrochemical NH_3_ generation [95]. The synthesis route started from the construction of Bi containing ZIF-8 by self-assembly under ambient conditions, and the Bi/NPC hybrid was yielded after the subsequent pyrolysis and phosphorating. Intriguingly, the N-doped carbon substrate was further modified instead of the formation of BiP. With the synergistic effects of Bi catalytic sites, conductive carbon supports, and P-dopant proton suppliers, superior catalytic performance toward NH_3_ synthesis was achieved with 13.58% FE at − 0.4 V versus RHE.

The structural tunability and synergistic effect of the bimetal-supported heteroatom-doped carbon catalyst can improve the performance of NRR compared with the single metal-based catalyst. Using ZIF-8 as a precursor, electro-rich metal sites can be obtained by thermolysis followed by acid etching. Ma et al. prepared an N-doped hollow carbon polyhedron supported by PdZn nanoparticles by etching, which can increase more electrons for vacancies in Pd-based catalysts and promote N_2_ adsorption, thus improving the performance of NRR [96]. The FE of NH_3_ reaches 16.9%, and compared with PdZn NP in buffer solution, the NH_3_ yield of the catalyst after etching is significantly higher (5.28 μg h^−1^ mg_cat._^−1^). Furthermore, Cong et al. synthesized a CoRu@NC after annealing the Ru@ZIF-67 that was generated through the ion-exchange reactions [97]. The CoRu@NC hybrids exhibited efficient electrocatalytic activity on NRR, providing an NH_3_ production rate of 56.82 μg h^−1^ mg_cat._^−1^ with a 2.02% FE at − 0.3 V versus RHE. In these works, the synergistic effect of bimetallic active sites was emphasized to be one of the vital factors for enhancing electrochemical N_2_ conversion.

Inspired by nitrogenase in nature, molybdenum and iron-based catalysts have drawn extensive attention in the field of NRR. The bimetallic NC nanomaterials from molybdenum or iron precursors show advanced characteristics that benefit the catalytic process, such as bimetallic active sites, synergistic effects, and excellent electrical conductivity. For instance, Zhang et al. prepared bimetallic nanoparticles of Mo-Co by pyrolysis of Mo-Co-ZIF-8 precursor. Compared with the single metal Co/NC, the catalytic activity and selectivity of bimetallic Mo-Co /NC are significantly enhanced, and the ammonia yield and FE are 89.8 µmol h^−1^ g_cat._^−1^ and 13.5%, respectively [98]. In addition, under the continuous electrolysis of 50000 s, the bimetallic Mo-Co /NC shows good electrochemical stability. Experimental studies have proved that pyridine nitrogen and pyrrole nitrogen are beneficial to NRR, to distinguish the ammonia generated from N_2_ rather than N in the precursor. Chen et al. synthesized a nitrogen-free catalyst and obtained porous microspheres of MoFe-PC by pyrolysis of phosphoric acid and bimetallic MOF, which not only included the bimetallic active site but also inherited the porous structure of the MOF precursor [99]. It is an efficient NRR catalyst with the highest ammonia yield of 34.23 µg h^−1^ mg_cat._^−1^ and FE of 16.83% at − 0.5 V versus RHE. Cu-Mo bimetallic MOFs were also used as the precursors for fabricating electrocatalysts for N_2_ reduction. Furthermore, Ma et al. constructed a composite material with MOF-derived Fe_2_O_3_ NPs anchored on MoS_2_ nanoflowers (Fe_2_O_3_@MoS_2_) and used it in the electrochemical N_2_ fixation as the catalyst [100]. The Fe_2_O_3_ NPs were synthesized through the calcination of Fe-MOF and subsequently confined by the in situ generated MoS_2_ nanoflowers. Compared to the MoS_2_ nanoflowers, enhanced NRR catalytic activity and stability were provided by the Fe_2_O_3_@MoS_2_ composite.

**Fig. 15:**
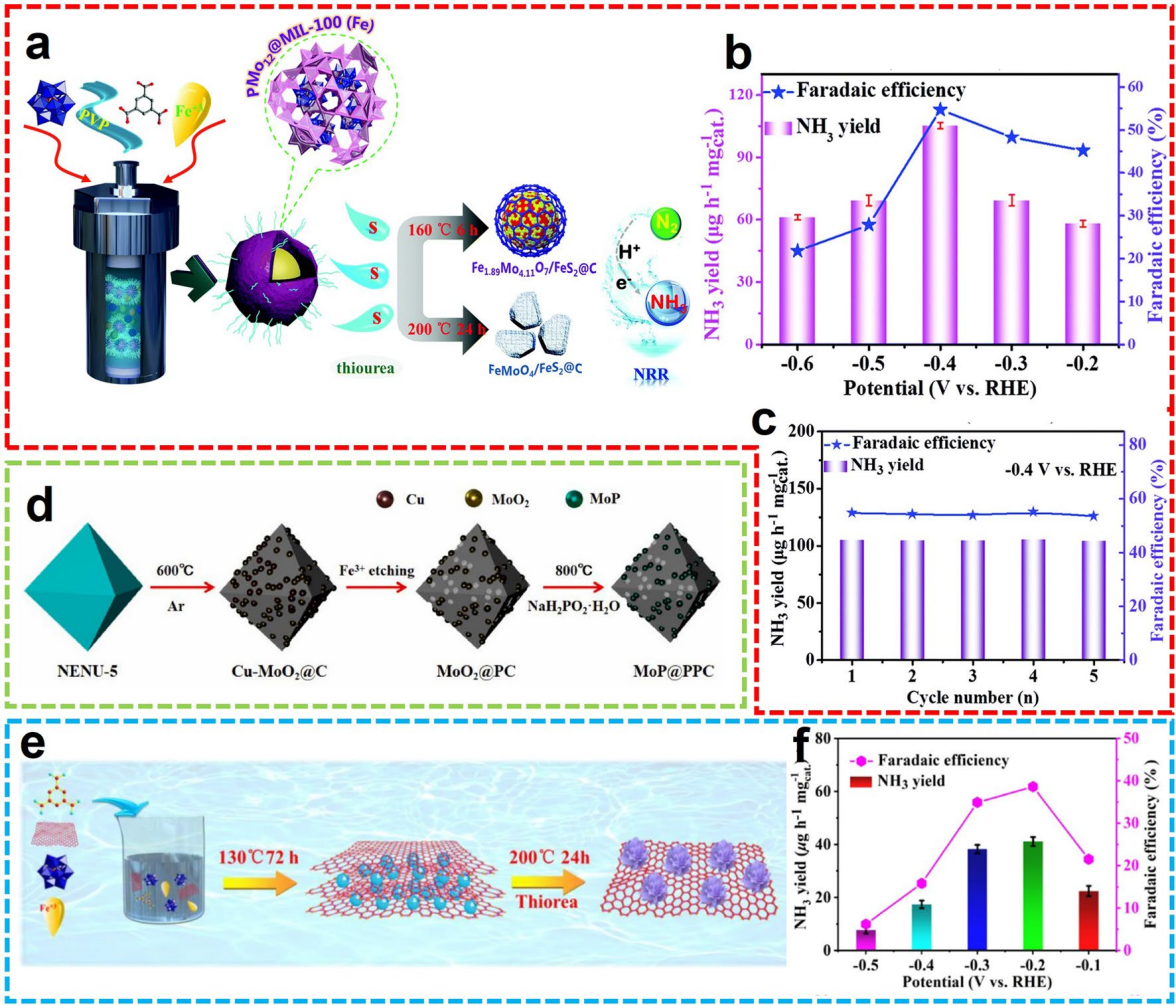
**a** Schematic illustration and **b**, **c** NRR performance of Fe_1.89_Mo_4.11_O_7_/FeS_2_@C. Reproduced with permission from Ref. [101]. Copyright 2020, Royal Society of Chemistry. **d** Schematic synthesis of MoP@PPC. Reproduced with permission from Ref. [102]. Copyright 2021, Elsevier. **e** Schematic synthesis, **f** NRR performance of FeS_2_/MoS_2_@RGO. Reproduced with permission from Ref. [103]. Copyright 2022, Elsevier

Wang et al. designed an electrocatalyst for coupling Fe and Mo as the active component, selected polyoxometalate-based MOF with polyvinylpyrrolidone (PMo_12_@MOF-100(Fe)@PVP) as the precursor, using their multi-component and multi-interface structure to induce electron transfer and improve the electrical conductivity of the hybrid material (Fig. 15a) [101]. The authors reported for the first time a POMOFs-derived Fe_1.89_Mo_4.11_O_7_/FeS_2_@C catalyst with acidic potassium sulfate as an electrolytic solution, the FE of Fe_1.89_Mo_4.11_O_7_/FeS_2_@C is as high as 54.7%, with an NH_3_ yield rate of 105.3 μg h^−1^ mg_cat._^−1^ (Fig. 15b, c). In another example, Duan and co-workers developed MoP NPs implanted in P-doped porous carbon (MoP@PPC) from PMo_12_-based MOF (NENU-5) after high-temperature pyrolysis, oxidative etching, and phosphating (Fig. 15d) [102]. The Cu nodes of the original MOF were removed during the etching process, thus generating phosphatized Mo dispersed on the P-doped carbon with the octahedral morphology. Afterward, another POMOF-derived for NH_3_ electrosynthesis was reported from the same group [103]. Based on the host–guest-assisted strategy, the nanostructured bimetallic sulfides, FeS_2_/MoS_2_@RGO were synthesized through the reaction between thiourea and pre-fabricated PMo_12_@MOF-100(Fe)@RGO (Fig. 15e). Due to the integration of electrochemical characteristics of individual components, the hybrid material displayed excellent catalytic performance for NRR, with optimized NH_3_ yield of 41.1 μg h^−1^ mg_cat._^−1^ and maximum FE of 38.6% at − 0.2 V versus RHE (Fig. 15f). The sulfide composite showed sufficient catalytic activity and stability in both acid and alkaline electrolytes, presenting great application value.

**Fig. 16:**
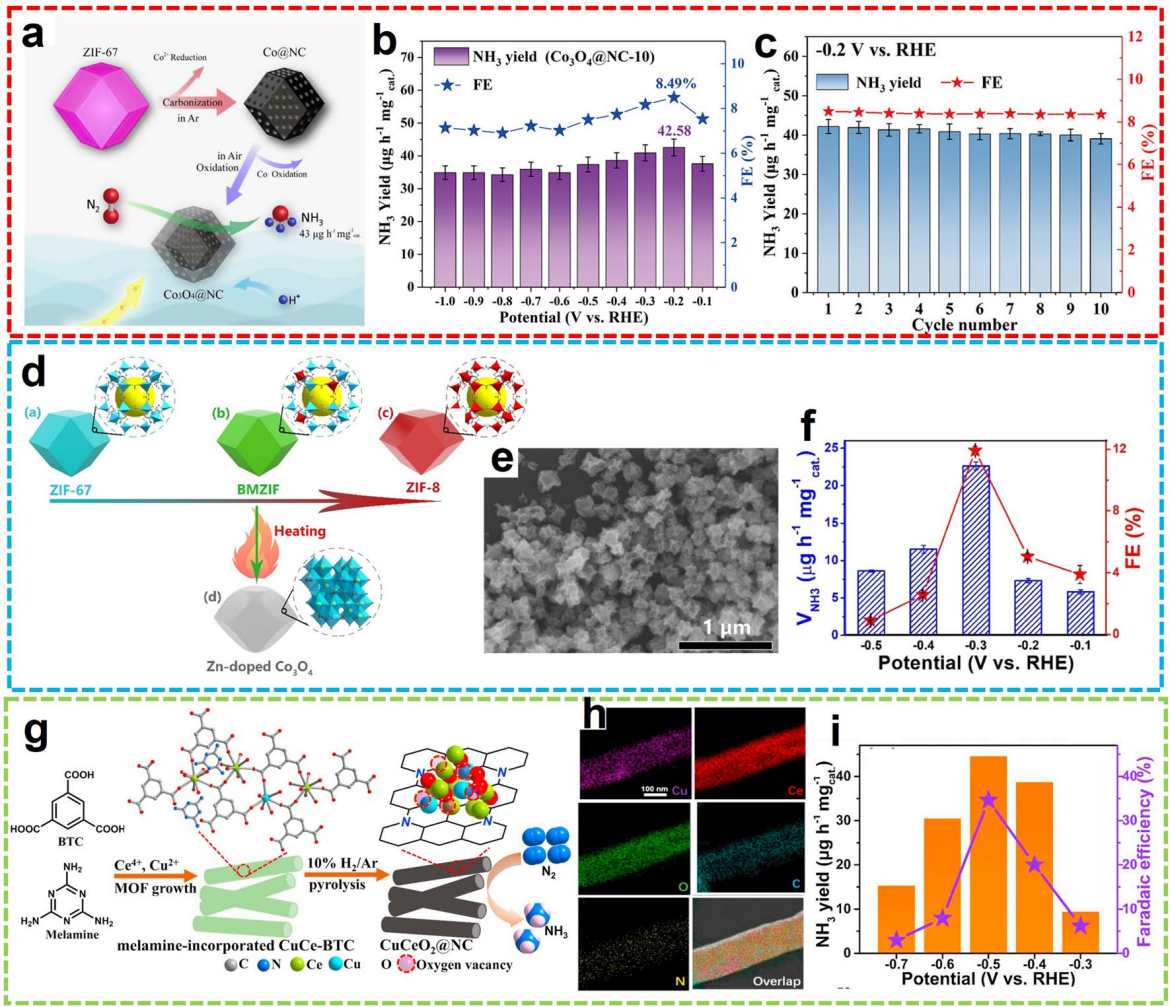
**a** Schematic illustration and **b**, **c** NRR performance of Co_3_O_4_@NC. Reproduced with permission from Ref. [107]. Copyright 2019, American Chemical Society. **d** Schematic synthesis, **e** SEM image, and **f** NRR performance of Zn-Co_3_O_4_. Reproduced with permission from Ref. [109]. Copyright 2021, American Chemical Society. **g** Schematic synthesis, **h** HAADF-STEM element mapping images, and **i** NRR performance of CuCeO_2_@NC. Reproduced with permission from Ref. [110]. Copyright 2021, Elsevier

Transition metal oxides (TMOs) are also promising substitutes for NRR because of their earth abundance and low price [104]. MOF-derived TMOs have attracted extensive attention in the field of catalysis. For early stage transition metals, the C-doped TiO_2_/C (C-Ti_*x*_O_*y*_/C) and C/Y-stabilized ZrO_2_ derived from related MOFs were fabricated for the electrochemical reduction of N_2_ to NH_3_ under mild conditions [105, 106]. For iron group elements, the MOF-derived Co_3_O_4_@NC nanocomposites were reported by Luo et al. with high specific surface products and structural stability (Fig. 16a) [107]. Co_3_O_4_ with oxygen vacancy (V_o_) was obtained by pyrolysis, and the synergistic effect with NC improved the performance of NRR. In 0.05 M H_2_SO_4_, it exhibits a high NH_3_ yield of 42.58 μg h^−1^ mg_cat._^−1^ with 8.5% FE at − 0.2 V versus RHE (Fig. 16b). During the 6 cycles of electrocatalysis, the Co_3_O_4_@NC catalytic stability was ensured with neglectable fluctuation (Fig. 16c). Afterward, the MOF-derived hollow C@NiO@Ni catalyst was developed by the same group, exhibiting excellent NRR performance in 0.1 M KOH with a high NH_3_ yield of 43.15 μg h^−1^ mg_cat._^−1^ and FE of 10.9% at − 0.7 V versus RHE [108]. Similarly, Wen and co-workers developed Zn-doped Co_3_O_4_ nano-polyhedrons for the electrochemical N_2_ fixation (Fig. 16d) [109]. After low-temperature oxidation of the Zn/Co bimetallic ZIF precursors, the morphology of ZIF was retained with abundant simultaneously formed V_o_ that acted as the Lewis acid sites (Fig. 16e). With the synergistic effect of V_o_ and electron-rich Co sites, the N_2_ transformation was promoted, offering an NH_3_ yield of 22.71 μg h^−1^ mg_cat._^−1^ with an 11.9% FE (Fig. 16f). In another case, the promotive effect of V_o_ for electrocatalysis was further verified by Cu-doped CeO_2_ NPs on carbon nitride support (CuCeO_2_@NC) [110]. Ye et al. fabricated the CuCeO_2_@NC through the annealing of melamine-cooperated CuCe-BTC under a reducing atmosphere (Fig. 16g, h). The maximum FE of 34.6% was provided with a high NH_3_ yield of 44.5 μg h^−1^ mg_cat._^−1^ (Fig. 16i). Moreover, the N_2_ fixation activity was further improved by introducing urea in the neutral electrolytic solution, using the MOF-derived CuO/Cu_2_O@CD-CN/NiF as the electrocatalyst [111]. According to these advances in MOF-derived TMO catalysts for NRR, the V_o_ was claimed as the indispensable catalytic site.

In addition to TMO, other transition metal complexes (phosphide, sulfide) have also been widely used in electrocatalysis. For example, Wei et al. reported that ZIF-67-derived cobalt disulfide nanoparticles supported in nitrogen-doped carbon (CoS_2_@NC) for NH_3_ synthesis [112]. With excellent catalytic activity and electrical conductivity, CoS_2_@NC catalyzed artificial N_2_ fixation provided a large NH_3_ yield of 17.45 μg h^−1^ mg_cat._^−1^ and a high FE of 4.6% at − 0.15 V versus RHE in 0.1 M HCl. Furthermore, Liu et al. developed a highly selective FeNi_2_S_4_/NiS electrocatalyst prepared by electrodeposition of Fe/Ni MOF-74 on carbon cloth as a precursor and curing at high temperatures [113]. The exposed crystal surface of the catalyst and the electronic structure of the d-band of the catalyst was adjusted by changing the stoichiometric ratio of Fe: Ni to enhance the selective adsorption of N_2_. Thus, heterogeneous interfaces with dual activity, which have stronger NRR performance than single FeS_2_ or NiS, exhibit ultra-high NH_3_ yields up to 128.398 ± 1.32 μg h^−1^ cm^−2^ and a high FE of 28.64 ± 0.18% at − 0.30 V versus RHE in alkaline electrolytic solutions. In another case, Wu et al. synthesized NiCoS/C nanocages with enhanced chemical coupling using ZIF-67 as a precursor and applied them to the reduction of N_2_ [114]. DFT calculations demonstrated that the strong coupling between C and NiCoS played an important role in reducing NRR overpotential and improving NRR selectivity. In 0.1 M Li_2_SO_4_, the peak ammonia production reached 58.5 μg h^−1^ mg_cat._^−1^.

For metal phosphides, Guo et al. prepared a novel MOF of cobalt phosphide hollow nanocages assembled from CoP nanosheets [115]. This nanoparticle-nanosheet-nanocages hierarchical structure provides abundant active sites for NRR, with an FE of 7.36% at 0 V and maximum NH_3_ yields of 10.78 µg h^−1^ mg_cat._^−1^ at − 0.4 V versus RHE. Li and co-authors reported a nitrogen-doped carbon nanosheet embedded with Cu_3_P clusters (Cu_3_P/NC) for the electrochemical fixation of N_2_ [116]. The hybrid was synthesized through a low-temperature pyrolysis-phosphating strategy, with Cu-MOF precursors. Benefiting from the N-dopants that facilitated the electrical conductivity and defect formation as well as the phosphate that tuned the d-band of Cu, the generation of NH_3_ was achieved with a 10.4 yield rate and 6.3% FE at − 0.1 V versus RHE. As a result, the green and efficient electrochemical NRR to NH_3_ products were further enriched with these advances in MOF-derived metal phosphides.

In all, MOF-derived nanostructures and their hybrids were able to be fabricated rationally and precisely with the merits of adjustable structures, abundant compositions, and diverse morphologies originating from the MOF precursors. To date, these materials are undoubtedly one of the promising candidates for electrocatalytic NH_3_ synthesis under mild conditions. The related catalysts and their performance are listed in Table 2.

**Table 2:** The performance of MOF-derived electrocatalysts in NRR

Material	Precursor (MOF)	Electrolytic solution	Ammonia yield	FE	Potential	Refs.
NPC-750	ZIF-8	0.05 M H_2_SO_4_	23.8 μg h^−1^ mg_cat._^−1^	1.42%	− 0.9 V versus RHE	[77]
C-ZIF-1100	ZIF-8	1 M KOH	57.8 μg h^−1^ cm^−2^	10.2%	− 0.3 V versus RHE	[78]
NP-C-MOF-5	MOF-5	0.1 M HCl	1.08 μg h^−1^ mg_cat._^−1^	0.52%	− 0.1 V versus RHE	[79]
S/N-MPC	MAF-5 & MAF-6	0.05 M H_2_SO_4_	45.51 μg h^−1^ mg_cat._^−1^ (− 0.3 V)	25.16% (-0.2 V)	− versus RHE	[80]
Ru SAs/N–C	ZIF-8	0.05 M H_2_SO_4_	120.9 μg h^−1^ mg_cat_.^−1^	29.6%	− 0.2 V versus RHE	[81]
Ru@ZrO_2_/NC	UiO-66	0.1 M HCl	4085 μg h^−1^ mg_cat._^−1^ (-0.21 V)	21% (-0.11 V)	− versus RHE	[82]
ISAS-Fe/NC	Fe-ZIF-8	0.1 M PBS	62.9 μg h^−1^ mg_cat._^−1^	18.6%	− 0.4 V versus RHE	[83]
Fe_1_-N–C	PCN-222(Fe)	0.1 M HCl	0.95 μg h^−1^ cm^–2^	4.51%	− 0.05 V versus RHE	[84]
Fe–N/C-CPs	ZIF-8	0.5 K_2_SO_4_ (pH = 3.5)	2.27 μg h^−1^ mg_cat._^−1^	7.67%	− 0.2 V versus RHE	[85]
CSA/NPC	ZIF-8/-67	0.05 M Na_2_SO_4_	14.45 μg h^−1^ cm^–2^	10.5%	− 0.2 V versus RHE	[86]
Co/N-doped C	ZIF-67	0.1 M KOH	5.1 μg h^−1^ mg_cat._^−1^ (-0.4 V)	10.1%(-0.1 V)	− versus RHE	[87]
Ni-N_*x*_-C	ZIF-8	0.1 KOH	85 μg h^−1^ cm^−2^ (-0.3 V)	22.2%	− 0.2 V versus RHE	[88]
NC/Bi SAs/TiN/CC	Bi-MOF	0.1 M Na_2_SO_4_	76.15 μg h^−1^ mg_cat._^−1^ (-0.8 V)	24.6% (-0.5 V)	− versus RHE	[89]
NC@Ru	ZIF-8	0.1 M KOH	16.68 μg h^−1^ mg_cat._^−1^ (− 0.4 V)	14.23% (− 0.3 V)	− versus RHE	[90]
Co/C-900	ZIF-67	0.1 M KOH	79.22 μg h^−1^ cm^–2^	11.53%	− 0.3 V versus RHE	[91]
Co@NC	ZIF-67	0.1 M Na_2_SO_4_	9.61 μg h^−1^ cm^–2^	21.79%	− 0.9 V vs Ag/AgCl	[92]
Fe–N/C-CNTs	ZIF-8	0.1 M KOH	34.83 μg h^−1^ mg_cat._^−1^	9.28%	− 0.2 V versus RHE	[93]
Bi NPs@CRs	Bi-BTC	0.1 M HCl	20.80 μg h^−1^ mg_cat._^−1^(-0.55 V)	11.50%(-0.6 V)	− versus RHE	[94]
Bi/NPC	Bi-ZIF-8	0.1 M KHCO_3_	3.12 μg h^−1^ cm^−2^ (-0.6 V)	13.58% (-0.4 V)	− versus RHE	[95]
Etched-PdZn/NHCP	ZIF-8	0.1 M PBS	5.28 μg h^−1^ mg_cat._^−1^	16.9%	− 0.2 V versus RHE	[96]
CoRu@NC	ZIF-67	0.1 M KOH	56.82 μg h^−1^ mg_cat._^−1^	2.02%	− 0.3 V versus RHE	[97]
Mo-Co/NC	MoCoZn-ZIFs	0.1 M Na_2_SO_4_	89.8 μmol h^−1^ g_cat._^−1^	13.5%	− 0.1 V versus RHE	[98]
MoFe-PC	Mo/Fe MOF	0.1 M HCl	34.23 μg h^−1^ mg_cat._^−1^	16.83%	− 0.5 V versus RHE	[99]
Fe_2_O_3_@MoS_2_	Fe-BDC	0.1 M Na_2_SO_4_	112.15 μg h^−1^ mg_cat._^−1^	8.62%	− 0.4 V versus RHE	[100]
Fe_1.89_Mo_4.11_O_7_/FeS_2_@C	MOF-100(Fe)	1.0 M K_2_SO_4_ (pH = 3.5)	105.3 μg h^−1^ mg_cat._^−1^	54.7%	− 0.4 V versus RHE	[101]
MoP@PPC	NENU-5	0.1 HCl	28.73 μg h^−1^ mg_cat._^−1^	2.48%	− 0.3 V versus RHE	[102]
FeS_2_/MoS_2_@RGO	MIL-100(Fe)	1.0 M K_2_SO_4_ (pH = 3.5)	41.1 μg h^−1^ mg_cat._^−1^	38.6%	− 0.2 V versus RHE	[103]
C-Ti_*x*_O_y_/C	MIL-125(Ti)	0.1 M LiClO_4_	14.8 μg h^−1^ mg_cat._^−1^	17.8%	− 0.4 V versus RHE	[105]
C/Y-stabilized ZrO_2_	Y-UiO-66	0.1 M Na_2_SO_4_	24.6 μg h^−1^ mg_cat._^−1^	8.2%	− 0.4 V versus RHE	[106]
Co_3_O_4_@NC	ZIF-67	0.05 M H_2_SO_4_	42.58 μg h^−1^ mg_cat._^−1^	8.5%	− 0.2 V versus RHE	[107]
C@NiO@Ni	Ni-MOF	0.1 M KOH	43.15 μg h^−1^ mg_cat._^−1^	10.9%	− 0.7 V versus RHE	[108]
Zn-Co_3_O_4_	CoZn-ZIF	0.1 M HCl	22.71 μg h^−1^ mg_cat._^−1^	11.9%	− 0.3 V versus RHE	[109]
Cu_0.1_CeO_2_@NC	CuCe-BTC	0.1 M Na_2_SO_4_	44.5 μg h^−1^ mg_cat._^−1^	34.6%	− 0.5 V versus RHE	[110]
CuO/Cu_2_O@CD-CN/NiF	Cu-MOF	1.0 M Na_2_SO_4_ with 0.5 M urea	102.2 μg h^−1^ cm^–2^	23.9%	− 0.4 V versus RHE	[111]
CoS_2_@NC	ZIF-67	0.1 M HCl	17.45 μg h^−1^ mg_cat._^−1^	4.6%	− 0.15 V versus RHE	[112]
FeNi_2_S_4_/NiS/CC	MOF-74	0.1 M KOH	129.72 μg h^−1^ cm^−2^	28.82%	− 0.3 V versus RHE	[113]
NiCoS/C	ZIF-67	0.1 M Li_2_SO_4_	26.0 μg h^−1^ mg_cat._^−1^	12.9%	0 V versus RHE	[114]
CoP HNC	ZIF-67	1 M KOH	10.78 μg h^−1^ mg_cat_.^−1^ (− 0.4 V)	7.36% (0 V)	− versus RHE	[115]
Cu_3_P@NC	Cu-MOF	0.1 M Na_2_SO_4_	10.4 μg h^−1^ mg_cat._^−1^	6.3%	− 0.3 V versus RHE	[116]

## MOF-Related Electrocatalysts for Nitrate Reduction Reactions

To kill two birds with one stone, the electrochemical reduction of nitrate (NO_3_^–^) is considered a promising strategy to remove NO_3_^–^-containing pollutants in water with the simultaneous NH_3_ synthesis under ambient conditions, which is also called electrochemical denitration [117, 118]. Although extensive investigations have been reported in this field, there are few examples were realized using MOF-based electrocatalysts under mild conditions. Due to the complexity of the plausible mechanism of electrochemical denitration, several advanced works were presented yielding N_2_ as the major product. For example, Sun and co-workers proposed an electrochemical conversion of NO_3_^−^ to N_2_, adopting a Fe–Ni bimetallic MOF-derived nanomaterial as the effective electrocatalyst [119]. Theoretically, further reduction and protonation are essential for the more valuable NH_3_ product. According to the discussion about the NRR process in the previous section, with porous structure and intrinsic hydrophobicity, MOFs were also presumed to have the ability to exhibit their superior catalytic activity and product selectivity for electrochemical conversion of NO_3_^−^ to NH_3_ process under ambient conditions. More recently, the electrochemical conversion of NO_3_^−^ to NH_3_ was extensively explored by MOF-related catalysts, and recent advances were listed as follows.

### MOF-Based Electrocatalysts

**Fig. 17:**
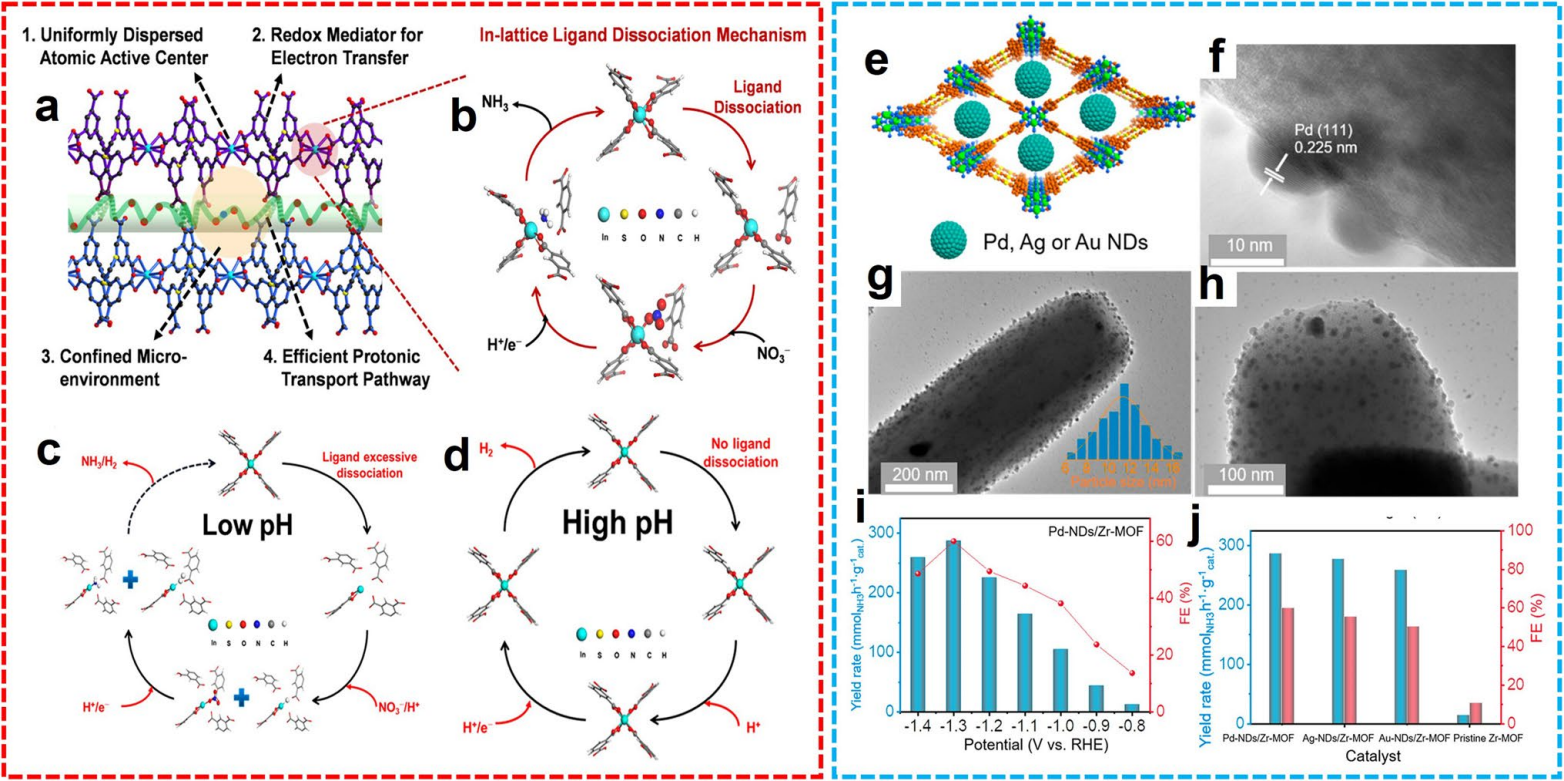
**a** Schematic illustration of the 2D In-MOF crystalline structure. Catalytic cycle of nitrate reduction **b** at pH =2 or 3, **c** at pH = 1, and **d** at pH = 4 or 5, respectively. Reproduced with permission from Ref. [120]. Copyright 2022, American Chemical Society. **e** Schematic structure of M-NDs/Zr-MOF. **f** HRTEM image, **g, h** TEM images, and **i** NO_3_RR performance of Pd-NDs/Zr-MOF. **j** Comparison of NH_3_ yields and FE values of different catalysts at -1.3 V vs. RHE. Reproduced with permission from Ref. [122]. Copyright 2022, American Chemical Society

Similar to the MOF-based electrocatalysts for NRR, the precisely designed and delicately synthesized pristine MOFs or MOF-hybrids with proper hydrophobicity, sufficient conductivity, and robust catalytic active sites can tune the NO_3_^−^ reduction for the harvest of NH_3_ products. For example, Lv et al. reported 2D In-MOF electrocatalysts with atomically dispersed active sites, high electron, and proton transport, and confined microporous environments for electrochemical denitration (Fig. 17a) [120]. After a thorough exploration of the catalysts and reaction conditions, the NH_3_ product was provided with the highest NH_3_ yield rate of 92.98 µg h^−1^ mg_cat._^−1^ (pH = 2) and optimal FE of 56.57% (pH = 3) at − 0.7 V versus RHE. Notably, the competing HER process can be tuned by the pH value of the electrolytic solution (Fig. 17b). With proper pH value, the metal active sites were properly exposed with appropriate ligand dissociation, while excessive ligand dissociation at low pH led to increasing water splitting (Fig. 17c) and no ligand dissociation at high pH caused complete cessation of NH_3_ generation (Fig. 17d). In another case that adopted 2D MOF, Zhu et al. developed a Cu cluster-modified conductive Cu-MOF composite electrocatalysts through in situ synthetic strategy for electrochemical reduction of NO_3_^–^ [121]. During the electrocatalysis, the pre-fabricated Cu-HHTP nanorods were partially transformed into metallic Cu clusters confined internally. In 0.5 M Na_2_SO_4_, 85.81% of NO_3_^−^ was converted into NH_3_ with 96.84% selectivity, a yield rate of 1.84 mg h^−1^ cm^−2^, and an FE of 67.55%. According to the DFT calculation, the metallic Cu filling inside MOF was identified as a catalytic active site and the catalytic performance was improved due to the facilitation of electron transfer by the Cu(111) crystal face. Furthermore, Jiang and co-workers fabricated a series of noble metal nanodots (NDs) encapsulated in Zr-MOFs (M-NDs/Zr-MOF, M = Pd, Ag, or Au) and applied them in the NO_3_RR process as the electrocatalysts (Fig. 17e-h) [122]. The NO_3_^−^ to NH_3_ catalytic activity was promoted by the confined metal NDs, while the conductivity and mass transport was facilitated with uniform-sized pores and redox-reversible tetrathiafulvalene (TTF) motifs inside the Zr-MOF skeleton. In 0.1 M Na_2_SO_4_ with 500 ppm NO_3_^−^, the highest NH_3_ yield was obtained as 287.31 µmol h^−1^ mg_cat._^−1^ with 58.1% FE at − 1.3 V versus RHE with Pd-NDs/Zr-MOF as the electrocatalyst (Fig. 17i, j). In another investigation using noble-metal-related MOF-hybrid electrocatalysts, Qin and co-workers reported the preparation of a Ru_*x*_O_*y*_ mosaic Ni-MOF composite (RuNi-MOF) and its application in electrochemical NO_3_RR [123]. Through solvothermal strategy, a nearly 100% production selectivity for NH_3_ was realized, with a maximum yield of up to 274 µg h^−1^ mg_cat._^−1^ and FE of ~ 73% at − 1.7 and − 1.2 V versus Ag/AgCl, respectively. According to the theoretical calculation, the Ru_3_ site was indicated as the main active center for the generation of NH_3_, undergoing a direct electron-mediated pathway.

### MOF-Derived Electrocatalysts

**Fig. 18:**
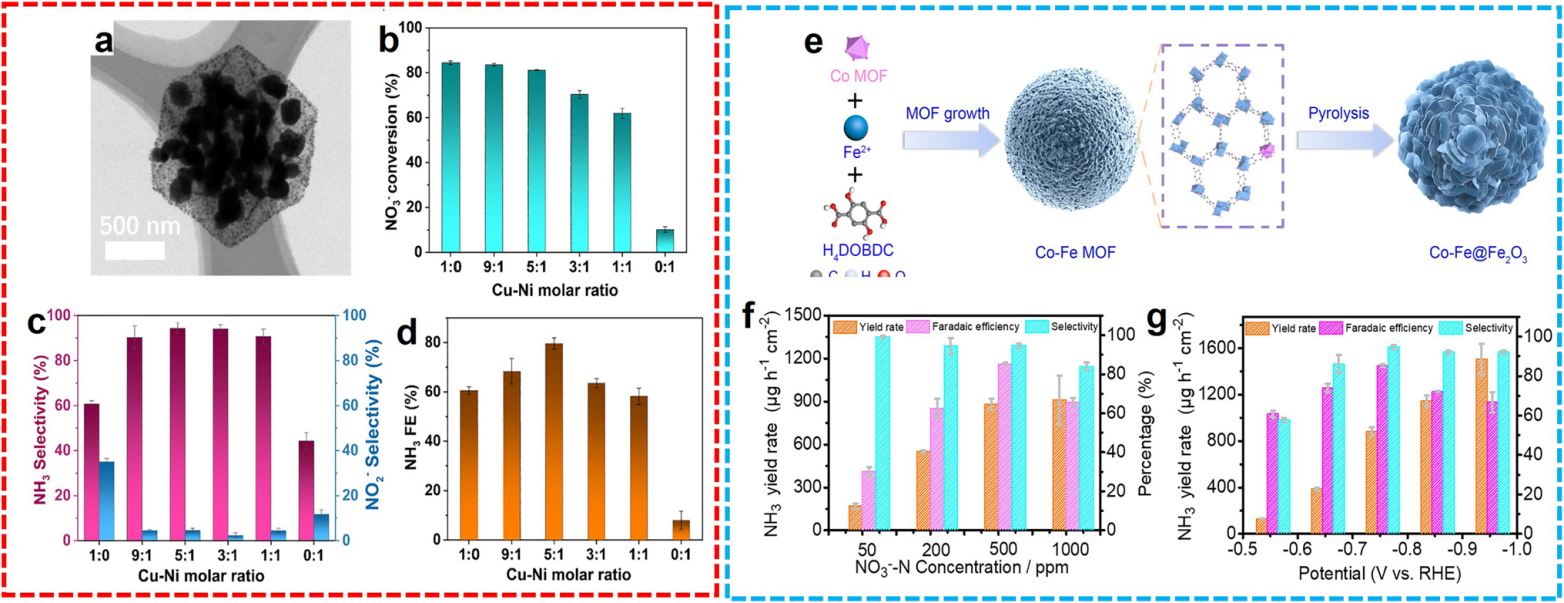
**a** TEM image of CuNi/NC-51. **b** Conversion rate of NO_3_^−^. **c** Selectivity of NH_3_ and NO_2_^−^. **d** FE of NH_3_ over CuNi/NC catalysts. Reproduced with permission from Ref. [126]. Copyright 2022, Elsevier. **e** Synthesis procedure of Co-Fe@Fe_2_O_3_. Comparison of NO_3_RR performance **f** at different NO_3_ concentrations and **g** at different potentials. Reproduced with permission from Ref. [127]. Copyright 2022, National Academy of Sciences

A pioneer work was reported by Li et al. during the investigation of the electrosynthesis of NH_3_ from NO_3_^−^ catalyzed by metal nanoclusters [124]. The authors prepared a series of pristine MOFs (Zn, Cu, Bi, Fe, and Co MOFs), MOF-derived NPC, MOF-derived SACs (Fe, Co, and Ni), and MOF-derived Co NPs/NPC and applied them in the electrochemical denitration. After extensive measurements, moderated catalytic performance was achieved in each case compared to that of strained Ru nanoclusters. Considering the relatively low cost of non-precious metal-based MOFs, this work inspired further exploration of the possibility of MOF-derived electrocatalysts in the NO_3_^−^-to-NH_3_ transformation process. Meanwhile, Zhu et al. developed a Cu–N-C electrocatalyst by pyrolysis of a modified Cu-MOF structure for the electrocatalytic denitration process [125]. The highly dispersed active Cu sites exhibited superior catalytic ability in the reduction of NO_3_^−^, alleviating the release of NO_2_^−^ during the reduction reaction. In 2022, Liu and colleagues developed bimetallic MOF-derived nitrogen-doped carbon materials equipped with CuNi alloy nanoparticles at the electrocatalysts for NO_3_RR [126]. With CuNi-BTC precursors prepared by the solvothermal method, a series of CuNi/NC with different Cu-Ni ratios were fabricated through pyrolysis. Inherited from the MOFs, the CuNi/NC catalysts maintained the octahedral morphology with the CuNi alloy particles enveloped in the nitrogen-doped carbon matrix (Fig. 18a). The conversion of NO_3_^−^ was higher than 80% with Cu/NC and decreased with the addition of Ni, while the values of NH_3_ selectivity and FE reached a peak at Cu: Ni = 5:1, obtaining 94.4% and 79.6%, respectively (Fig. 18b-d). A possible proton-mediated synthetic route of NH_3_ was proposed due to the synergistic effect of Cu-Ni for facilitating electron and proton transfer. Furthermore, Zhang et al. proposed a MOF-74-derived Co-doped Fe@Fe_2_O_3_ electrocatalyst for NO_3_RR (Fig. 18e) [127]. According to the experimental and computational results, the d-band of the Fe-center was modulated by the Co dopants, thus tuning the adsorption energy of intermediates and suppressing the HER process. The enhanced NH_3_ generation was shown with a maximum yield of 1505.9 μg h^−1^ cm^−2^ at − 0.95 versus RHE and 95.7% NH_3_ selectivity as well as FE of 85.8% at − 0.75 V versus RHE in 0.1 M Na_2_SO_4_ (Fig. 18f, g).

The study of MOF-related catalysts for electrochemical denitration is still in its infancy [128]. The inspiring works originated from the electrocatalytic reduction process of other molecules, such as carbon dioxide, oxygen, and nitrogen. It is attractive and challenging the investigation of precise design and synthesis of MOF-based or MOF-derived electrocatalysts with efficient catalytic activity and superior product selectivity. Moreover, since NH_3_ is valuable in modern industrial civilization and the possible mechanism of NO_3_RR is complicated, further exploration of the NH_3_ synthetic pathway with theoretical calculation and in situ characterization is essential for the guidance of practical use.

## Conclusion and Perspective

**Fig. 19:**
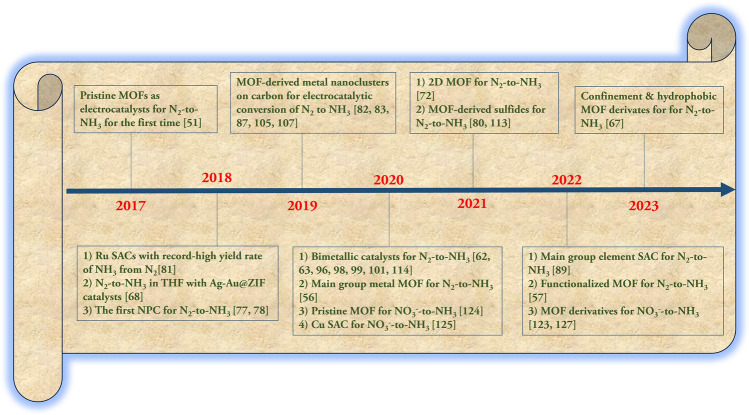
A timeline for electrosynthesis of ammonia catalyzed by MOF-related catalysts

In this review, we have summarized recent works focusing on the development of effective and efficient MOF electrocatalysts for electrochemical NH_3_ synthesis. A timeline since 2017 for selected works towards electrosynthesis of ammonia is shown in Fig. 19. According to our analysis of several recent studies, the electrocatalytic performance is determined by the ability of the superficial adsorption, reductive activation, and competitive NH_3_ selectivity that can be tuned by proper nanoengineering of the electrocatalysts. The electronic structure and morphology variation of the catalysts plays vital roles in achieving enhanced catalytic capability and superior product selectivity. Hence, fabricating electrocatalysts from MOFs indeed facilitates these electrochemical processes as practical and effective replacements for current industrial processes in synthetic and energy aspects. However, there are still drawbacks that are significant and challenging. First, the development of novel electrocatalysts is still dependent on experience or trial-and-error procedures, which costs a lot of time and energy. Second, the synthetic process and modification strategy often require harsh conditions to obtain the optimized morphology and boost catalytic performance, and reproducibility can be poor. Third, the surface environment may change under catalytic conditions, which can be difficult to predict and detect. Moreover, the N_2_ with its intrinsic insolubility and inertness requires extensive exploration to promote the reactivity, while the electrochemical conversion of nitrogen oxides is mainly valuable in waste gas and water treatment which is a drop in the ocean for modern ammonia needs. Therefore, some advanced techniques and methods are required to assist in the fabrication of high-performance MOF-related catalysts for NH_3_ synthesis in the future, as discussed below.

1. *Machine learning (ML)* is one of the significant breakthroughs in computer science these days. In the study of chemistry and material science, ML was adopted as a powerful tool to reasonably predict new molecules or materials for specific applications [129, 130]. With the help of an established database of nanomaterials, the proper candidates of electrocatalysts for ammonia production can be estimated before experimental corroboration. As a result, it should be feasible to develop functional NH_3_ synthesis catalysts from MOFs with the help of ML.
2. *Mild synthetic processing* is one of the ultimate pursuits in material science. Most of the current synthetic protocols for MOF-derived electrocatalysts in NH_3_ synthesis still require harsh conditions, excessive synthesis time and steps, and limited precursor types. Moreover, the existing synthetic protocols of robust MOF-related electrocatalysts are far from large-scale industrial applications. Novel strategies with simple manipulation and mild conditions are under investigation in the field of electrocatalysis [131]. Therefore, the development of new synthetic routes using new reaction precursors under mild conditions is important for the sustainable application of electrochemical NH_3_ production.
3. *In situ characterization* is a useful tool for understanding specific reaction mechanisms. Due to the complex and diverse reaction processes in the synthesis of NH_3_ from N_2_ or NO_3_^−^, in situ characterization should be helpful for the exploration of the reaction mechanism in detail. Through the in situ characterization, the key intermediates can be confirmed, the catalytic process can be understood, the conformational relationships can be clarified, and the synthesis of catalysts can be further guided. Thus, the development of MOF-related electrocatalysts with efficient and active catalytic performance toward the synthesis of NH_3_ can be extensively improved.
4. *Conversion of other N-containing substrates* is also an attractive research field in the electrosynthesis of ammonia. As nitrogen element is fully oxidized in NO_3_^−^ ion, other nitrogen oxides with relatively lower oxidation states have a high probability of being reduced to ammonia. There are existing developments of metal-based nanomaterials with extraordinary catalytic performance in the conversion of NO_2_^−^ [132–136] and prediction of 2D MOF-catalyzed electrochemical conversion of NO-to-NH_3_ through DFT calculation [137]. The MOF-based and derived materials are very promising catalysts for promoting the conversion of these NO_*x*_ particles or ions into NH_3_.

In all, the investigation of NH_3_ electrosynthesis under mild conditions is still at a preliminary stage and far from meeting the actual needs [138]. Developing comprehensive performance and low-cost electrocatalysts is no doubt the most promising outlet for constructing NH_3_ electrosynthesis that can complement or even replace the existing industrial processes. We hope that this review is able to offer inspiration for the rational development of MOF-related electrocatalysts for NH_3_ synthesis that would also make significant contributions and breakthroughs in materials science, synthetic chemistry, catalysis, and other fields.
